# Supplementary figures and images for: SIFa peptidergic neurons orchestrate the internal states and energy balance of male Drosophila melanogaster
Source: PLoS Biol. 2025 Sep 4;23(9):e3003345. doi: 10.1371/journal.pbio.3003345 (PMC12410877; doi:10.1371/journal.pbio.3003345)

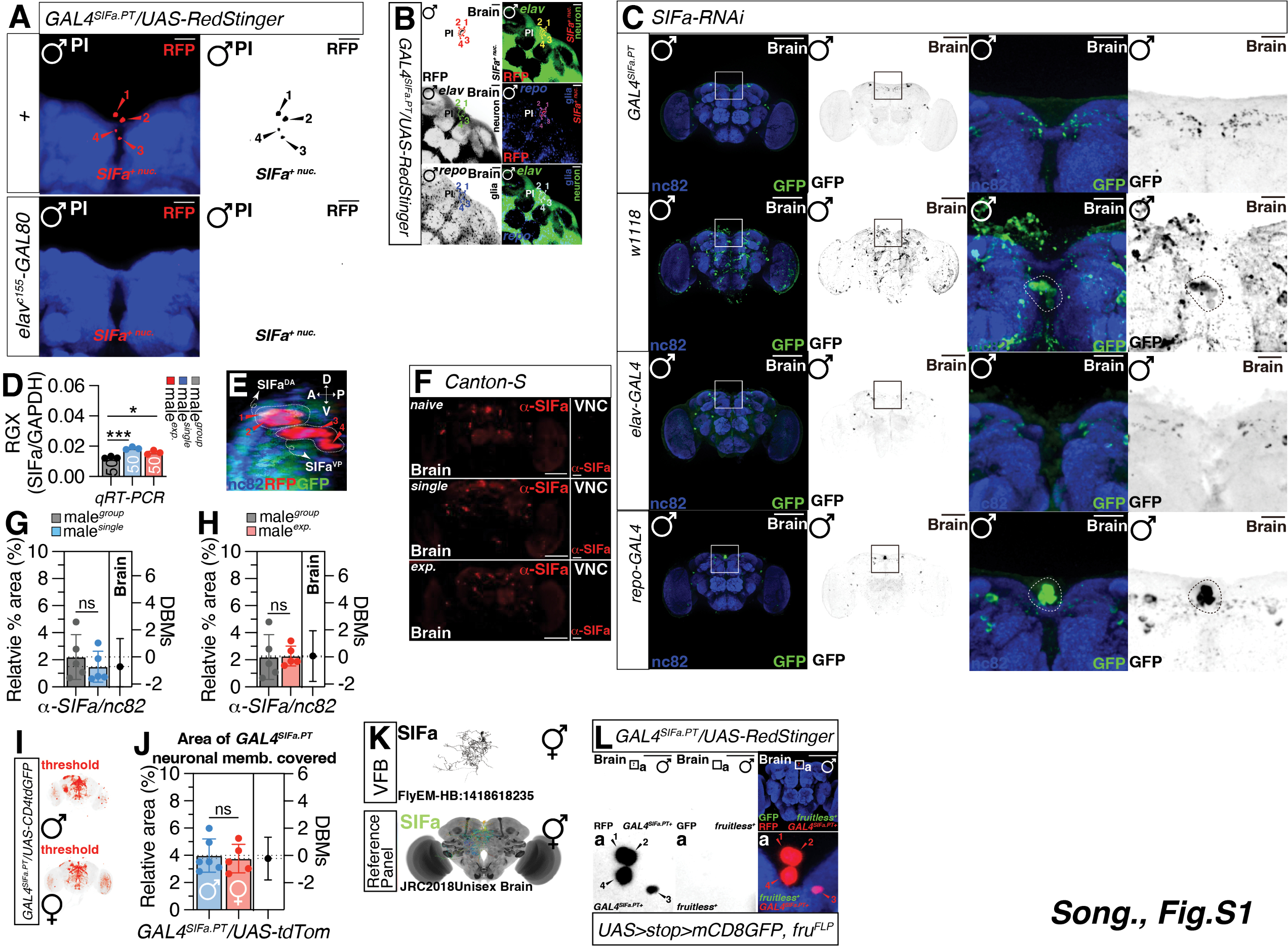

Supplement: S1 Fig — (A) Flies expressing GAL4SIFa.PT/+(top) or elav-GAL80; GAL4SIFa.PT drivers(bottom) together with UAS-RedStinger were immunostained with anti-SIFa (red) antibodies. Red arrowheads indicate SIFa region labeled by anti-SIFa antibodies. The right panels are presented as a gray scale to clearly show the axon expression patterns of SIFa neurons in the adult brain labeled by GAL4 drivers. Scale bars represent 10 µm. (B) Flies expressing GAL4SIFa.PT together with UAS-RedStinger were immunostained with anti-elav (green), anti-repo (blue) and anti-SIFa(red) antibodies. Arrowheads: cell body locations. Scale bars represent 10 µm. (C) Flies expressing SIFa-RNAi with GAL4SIFa.PT or w1118, elavc155 and repo-GAL4 drivers (from top to bottom) were immunostained with anti-SIFa (green) antibodies. The right panels are presented as a gray scale to clearly show the axon expression patterns of SIFa neurons in the adult brain labeled by GAL4 drivers. Scale bars represent 10 µm. (D) qRT-PCR results show that SIFa expression level in group (gray), single (blue) and exp (red) conditions. The y-axis depicts the relative expression level of SIFa, normalized to the CT value of the GAPDH gene. “RGX” denotes relative gene expression. See the Materials and methods for a detailed description of the Quantitative RT-PCR used in this study. Statistical significance determined by one-way ANOVA followed by Tukey’s comparisons test. **p < 0.01 (one-way ANOVA, F = 26.09, R2 = 0.8969; Tukey’s post-hoc). The ns represents non-significant differences. Sample sizes (n) are indicated in the figure panels. (E) SIFa neurons are categorized into two subpopulations, the anterior-dorsal SIFa neurons (SIFaDA) and the posterior-ventral SIFa neurons (SIFaVP), based on their anatomical positioning and putative functional roles. D: dorsal; V: ventral; A: anterior; P: posterior. Scale bars represent 10 µm. (F) The brains of CS Drosophila (left panel) and the VNC (right panel) were subjected to immunostained with anti-SI [file pbio.3003345.s002.tif]

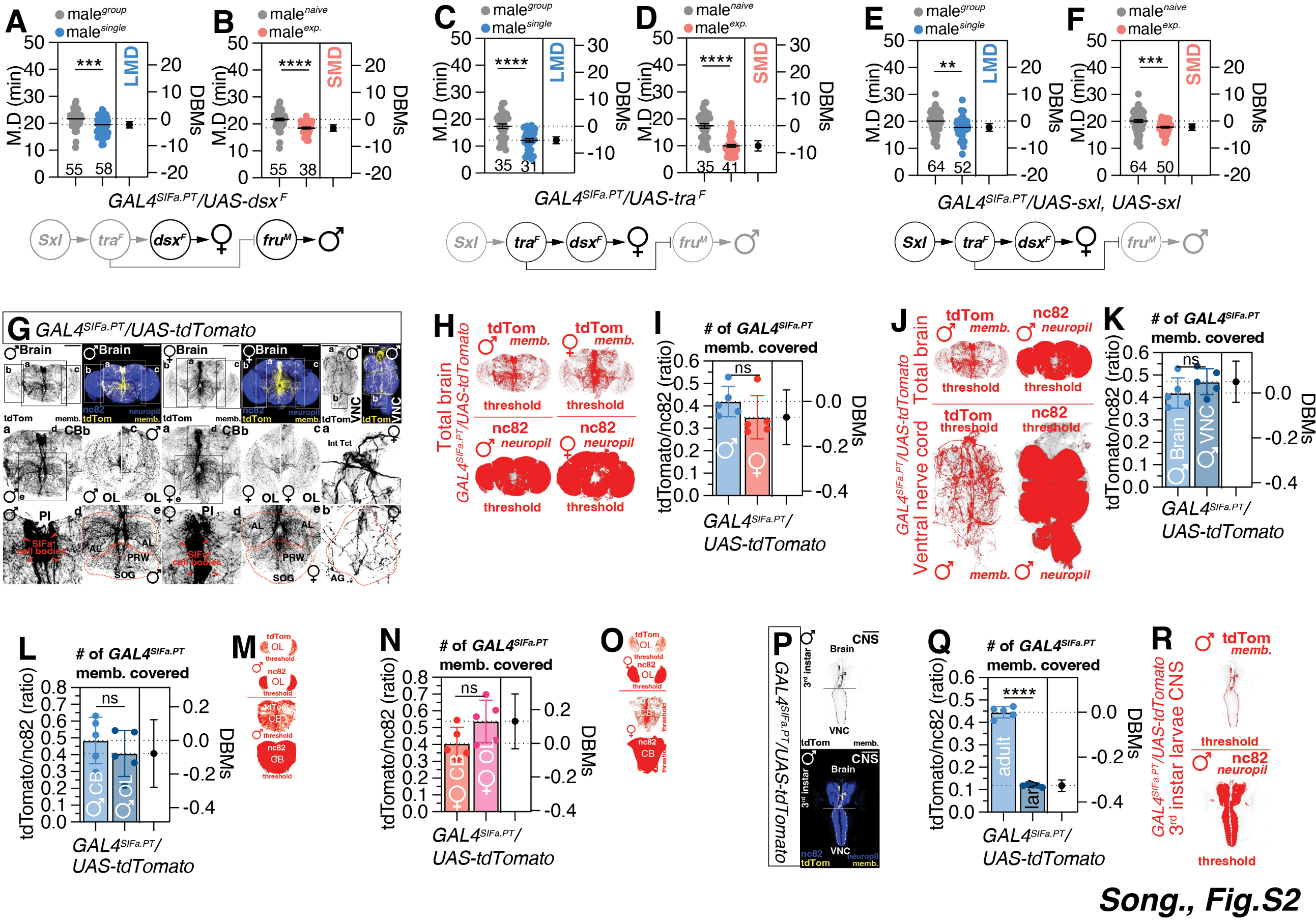

Supplement: S2 Fig — (A–F) MD assays for GAL4SIFa.PT drivers mediated expression of female form of doublesex (UAS-dsxF), transformer (UAS-traF), or sex lethal (UAS-sxl). (G) Flies expressing GAL4SIFa.PT drivers together with UAS-tdTomato were immunostained with anti-RFP (yellow), and nc82 (blue) antibodies. Areas outlined by boxes are enlarged in the bottom panel, respectively. Arrowheads: cell body locations. Scale bars represent 100 µm. (H, I) Quantification of RFP fluorescence in the male (left) and female (right) fly brain expressing GAL4SIFa.PT driver together with UAS-tdTomato. (H) The threshold of RFP fluorescence (upper panel), nc82 (bottom panel) in male and female fly brain was marked by threshold function of ImageJ. (I) Quantification of RFP fluorescence in male (blue) and female (pink) brain (two-tailed unpaired t test). In all plots and statistical tests. Data are presented as mean ± s.e.m. ns = not significant (p > 0.05), *p < 0.05, **p < 0.01, ***p < 0.001, ****p < 0.0001. The same symbols for statistical significance are used in all other figures. See the Materials and methods for a detailed description of the colocalization analysis used in this study. (J, K) Quantification of RFP fluorescence in brain and VNC of male fly (two-tailed unpaired t test). In all plots and statistical tests. Data are presented as mean ± s.e.m. ns = not significant (p > 0.05), *p < 0.05, **p < 0.01, ***p < 0.001, ****p < 0.0001. (L, M) Quantification of RFP fluorescence of CB and OL area in male (L, M) and female (N, O) (two-tailed unpaired t test). In all plots and statistical tests. Data are presented as mean ± s.e.m. ns = not significant (p > 0.05), *p < 0.05, **p < 0.01, ***p < 0.001, ****p < 0.0001. (P) Larva expressing GAL4SIFa.PT drivers together with UAS-tdTomato were immunostained with anti-RFP (yellow), and nc82 (blue) antibodies. Scale bars represent 100 µm. (Q) Quantification of the RFP fluorescence in third instar larvae and adult (two-tailed unpaired t test). In all plots and st [file pbio.3003345.s003.tif]

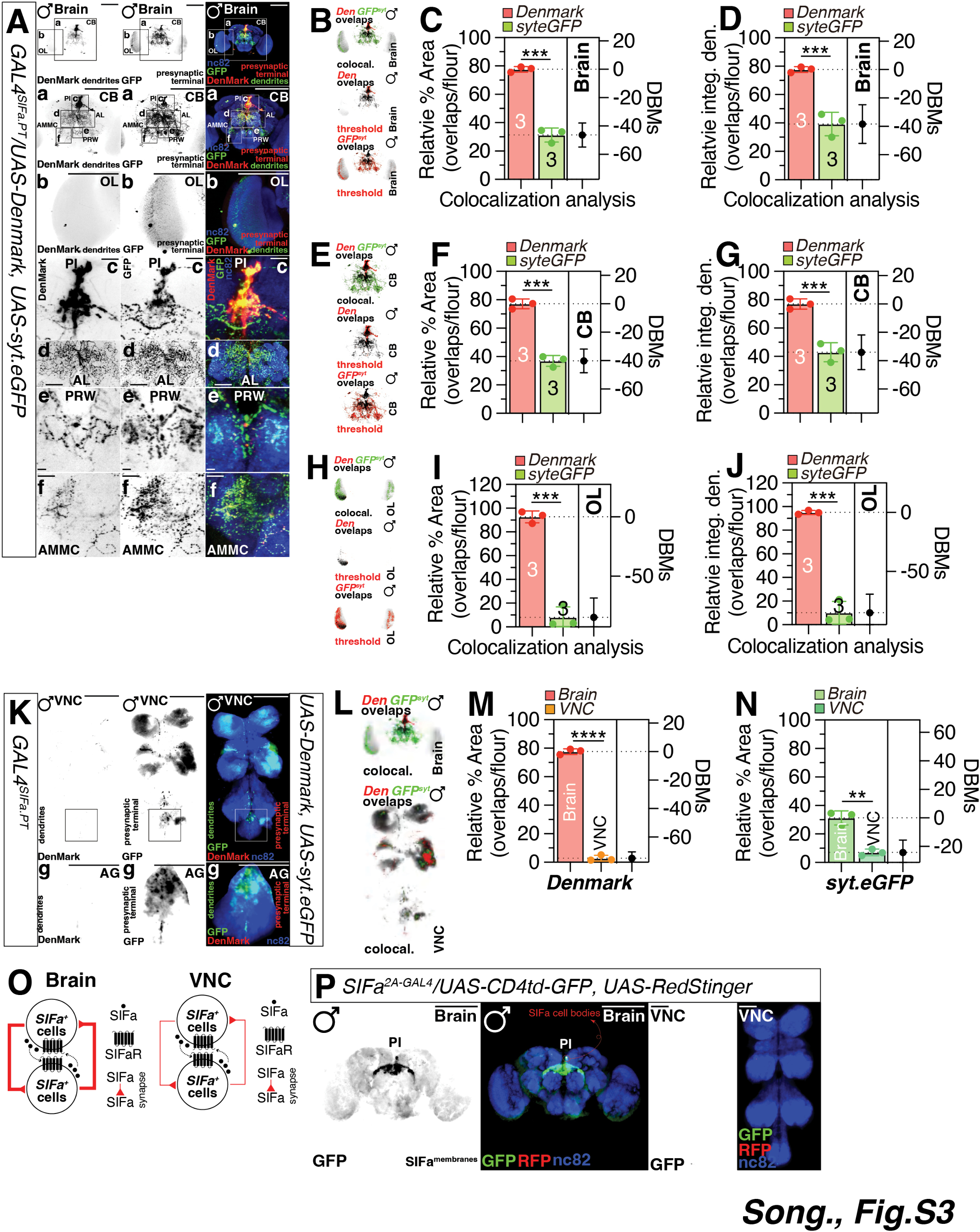

Supplement: S3 Fig — (A and K) Distribution of dendrites and presynaptic terminals of neurons labeled via GAL4SIFa.PT in the brain and VNC. Flies expressing GAL4SIFa.PT together with UAS-Denmark, UAS-syt.eGFP were immunostained with anti-GFP (green), anti-DsRed (red), and nc82 (blue) antibodies. Areas outlined by boxes are enlarged in bottom panel. Scale bars represent 100 μm in A and 50 μm in K. (B–J) Colocalization analysis of dendritic and presynaptic terminals of neurons labeled via GAL4SIFa.PT in the brain (B–D), CB (E–G), and OL (H–J) (two-tailed unpaired t test). In all plots and statistical tests. Data are presented as mean ± s.e.m. ns = not significant (p > 0.05), *p < 0.05, **p < 0.01, ***p < 0.001, ****p < 0.0001. Sample sizes (n) are indicated in the figure panels. See the Materials and methods for a detailed description of the colocalization analysis used in this study. (L–N) Quantification of dendritic and presynaptic terminals of neurons labeled via GAL4SIFa.PT in the brain and VNC (two-tailed unpaired t test). In all plots and statistical tests. Data are presented as mean ± s.e.m. ns = not significant (p > 0.05), *p < 0.05, **p < 0.01, ***p < 0.001, ****p < 0.0001. Sample sizes (n) are indicated in the figure panels. (O) Schematic shows that SIFa+ cells form extensive synapses each other within the brain but not in the VNC. (P) Male flies expressing SIFa2A-GAL4 together with UAS-CD4tdGFP and UAS-RedStinger were immunostained with anti-GFP (green), anti-RFP (red), and nc82 (blue) antibodies. Scale bars represent 100 µm. Underlying data for all graphs can be found in file S1 Data. (TIF) [file pbio.3003345.s004.tif]

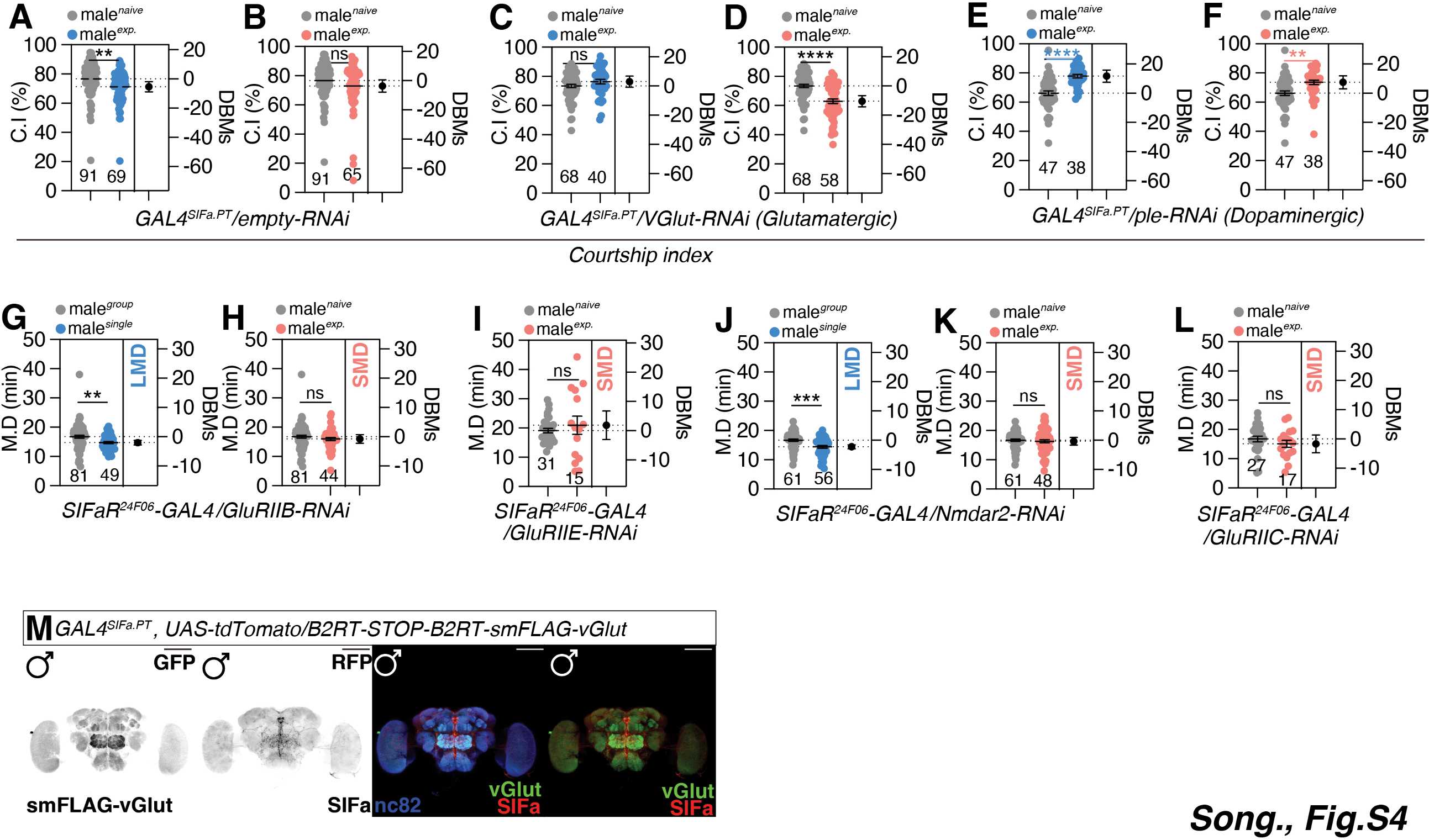

Supplement: S4 Fig — (A, B) courtship index of control flies in group, single, and exp conditions. MD assays for GAL4SIFa.PT mediated knockdown via empty-RNAi (two-tailed unpaired t test). In all plots and statistical tests. Data are presented as mean ± s.e.m. ns = not significant (p > 0.05), *p < 0.05, **p < 0.01, ***p < 0.001, ****p < 0.0001. Sample sizes (n) are indicated in the figure panels. (C–F) Courtship index of flies for GAL4-mediated knockdown of VGlut (Glutamatergic), and Ple (Dopaminergic) via VGlut-RNAi and ple-RNAi using the GAL4SIFa.PT (two-tailed unpaired t test). In all plots and statistical tests. Data are presented as mean ± s.e.m. ns = not significant (p > 0.05), *p < 0.05, **p < 0.01, ***p < 0.001, ****p < 0.0001. Sample sizes (n) are indicated in the figure panels. (G–L) MD assays for GAL4-mediated knockdown of GluRIIB(G-H), GluRIIE(I), Nmdar2(J-K) and GluRIIC(L) using SIFaR24F06-GAL4 driver (two-tailed unpaired t test). In all plots and statistical tests. Data are presented as mean ± s.e.m. ns = not significant (p > 0.05), *p < 0.05, **p < 0.01, ***p < 0.001, ****p < 0.0001. Sample sizes (n) are indicated in the figure panels. (M) Control of the Fig 2P. Fly’s brain after expression of the B2 recombinase in neurons of SIFa, Prior to excision of the STOP cassette smFLAG-vGlut is not expressed, above flies with UAS-mCD8RFP were immunostained with anti-DsRed (red), anti-FLAG (green), and nc82 (blue) antibodies. Scale bars represent 100 mm. Underlying data for all graphs can be found in file S1 Data. (TIF) [file pbio.3003345.s005.tif]

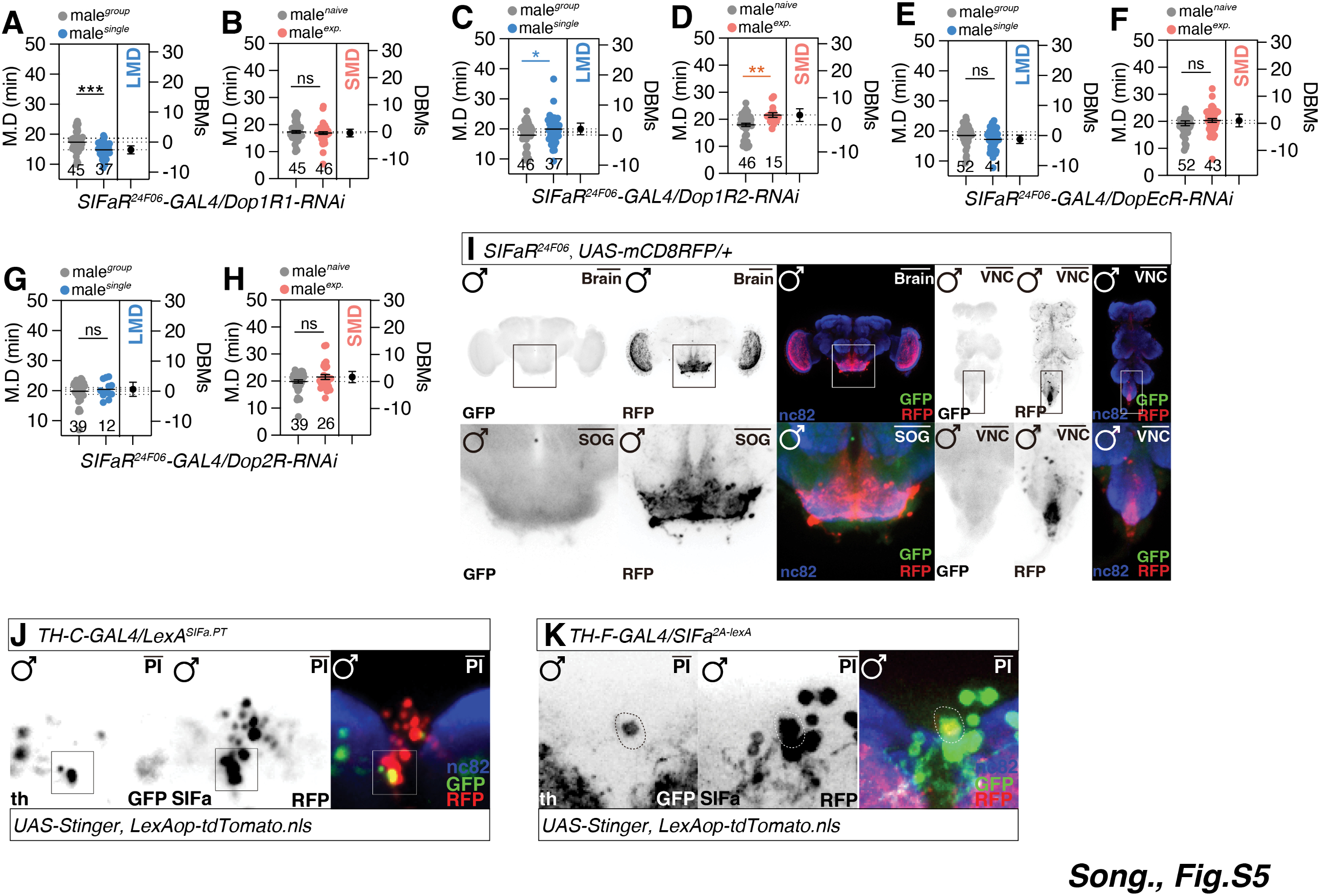

Supplement: S5 Fig — (A–H) MD assays of flies expressing the SIFaR24F06 driver together with (A, B) Dop1R1-RNAi, (C, D) Dop1R2-RNAi, (E, F) DopEcR-RNAi, (G, H) Dop2R-RNAi (two-tailed unpaired t test). In all plots and statistical tests. Data are presented as mean ± s.e.m. ns = not significant (p > 0.05), *p < 0.05, **p < 0.01, ***p < 0.001, ****p < 0.0001. Sample sizes (n) are indicated in the figure panels. (I) Control for Fig 3A. Male flies expressing SIFaR24F06 drivers together with UAS-mCD8RFP were imaged live under a fluorescent microscope with anti-GFP (green), anti-DsRed (red), and nc82 (blue) antibodies. Areas outlined by boxes are enlarged in the bottom panel. Scale bars represent 100 μm. (J) Flies expressing LexASIFa.PT with TH-C-GAL4 drivers together with UAS-stinger, LexAop-tdTomato.nls was immunostained with anti-GFP (green), anti-RFP (red), and nc82 (blue) antibodies. Scale bars represent 100 µm. (K) Flies expressing LexA2A-LexA with TH-F-GAL4 drivers together with UAS-stinger, LexAop-tdTomato.nls was immunostained with anti-GFP (green), anti-RFP (red), and nc82 (blue) antibodies. Scale bars represent 100 µm. Underlying data for all graphs can be found in file S1 Data. (TIF) [file pbio.3003345.s006.tif]

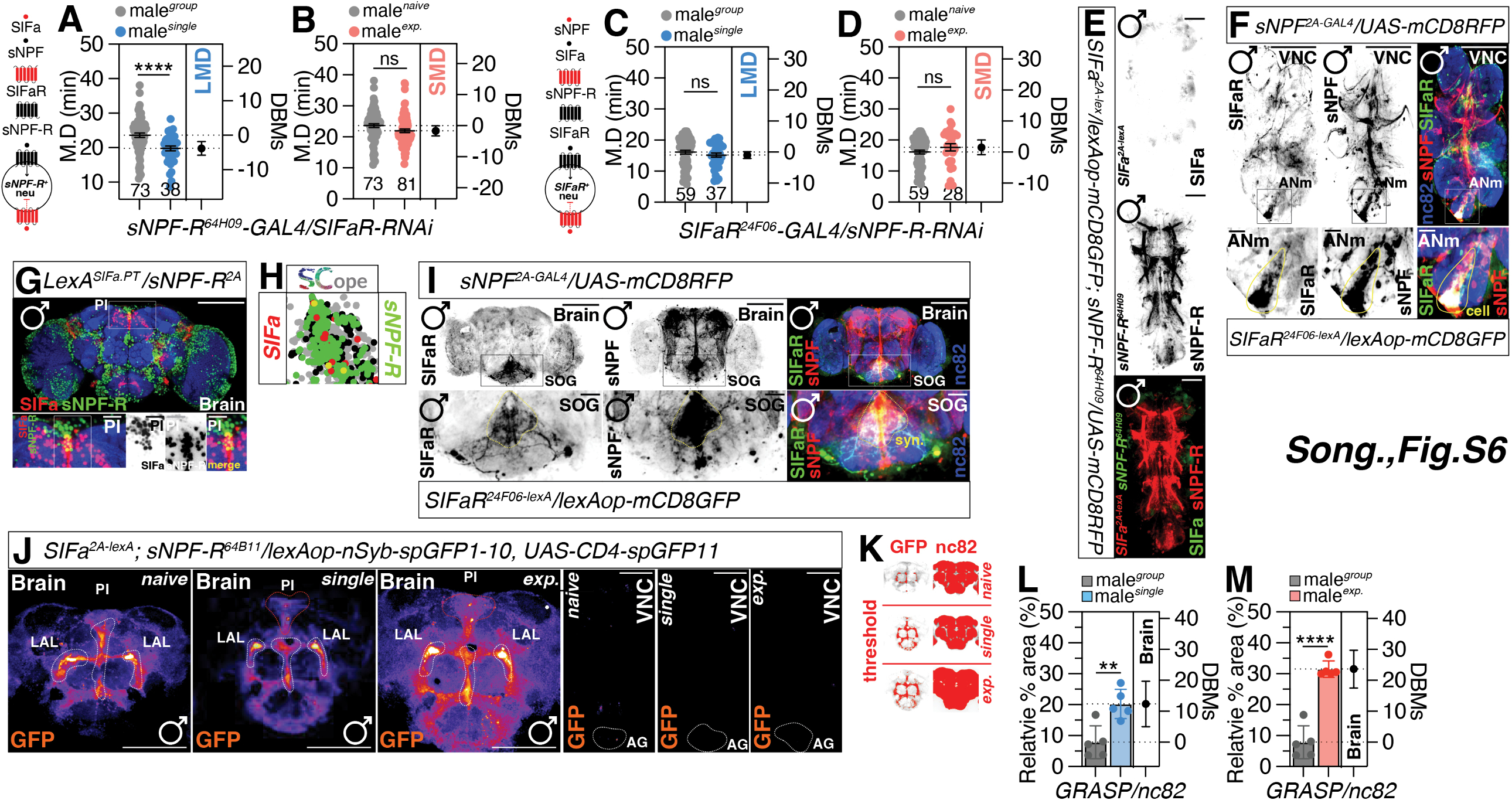

Supplement: S6 Fig — (A–D) MD assays for GAL4-mediated knockdown of SIFaR and sNPF-R via SIFaR-RNAi and sNPF-R-RNAi using the sNPF-R64H09-GAL4 driver (A, B), and SIFaR24F06-GAL4 driver (C, D) (two-tailed unpaired t test). In all plots and statistical tests. Data are presented as mean ± s.e.m. ns = not significant (p > 0.05), *p < 0.05, **p < 0.01, ***p < 0.001, ****p < 0.0001. Sample sizes (n) are indicated in the figure panels. (E) Male VNC of flies expressing sNPF-R64H09-GAL4 and SIFa2A-lexA drivers together with UAS-mCD8RFP and lexAop-mCD8GFP was immunostained with anti-GFP (green), anti-DsRed (red), and anti-nc82 (blue) antibodies. The top two panels are presented as a gray scale to clearly show the membrane expression patterns of SIFa+ neurons in the adult labeled by sNPF-R64H09-GAL4 driver. Scale bars represent 100 μm. (F, I) Male brain and VNC of flies expressing sNPF2A-GAL4 together with UAS-mCD8RFP was immunostained with anti-GFP (green), anti-DsRed (red), and anti-nc82 (blue) antibodies. Scale bars represent 100 μm. Areas outlined by boxes are enlarged in the bottom panel. Scale bars represent 50 μm. (G) Flies expressing LexASIFa.PT and sNPF-R2A-GAL4 drivers together with UAS-stinger, LexAop-tdTomato.nls was immunostained with anti-GFP (green), anti-RFP (red), and nc82 (blue) antibodies. Scale bars represent 100 µm. (H) Fly SCope single-cell RNA sequencing data of cells co-expressing SIFa together with sNPF-R. (J) GRASP assay for SIFa2A-lexA and sNPF-R64B11-GAL4 in male brain (left three columns) and VNC (right three columns). Male flies expressing SIFa2A-lexA, sNPF-R64B11-GAL4, and lexAop-nsyb-spGFP1-10, UAS-CD4-spGFP11 were dissected after 5 days of growth (mated male flies had 1 day of sexual experience with virgin females). The white dashed line highlights the GRASP signal. Scale bars represent 100 μm. (K–M) Quantification of synapses formed between SIFa2A-lexA and sNPF-R64H09-GAL4 in the brain. (K) The threshold of GFP fluorescence (left panel), nc82 (right panel) of naïv [file pbio.3003345.s007.tif]

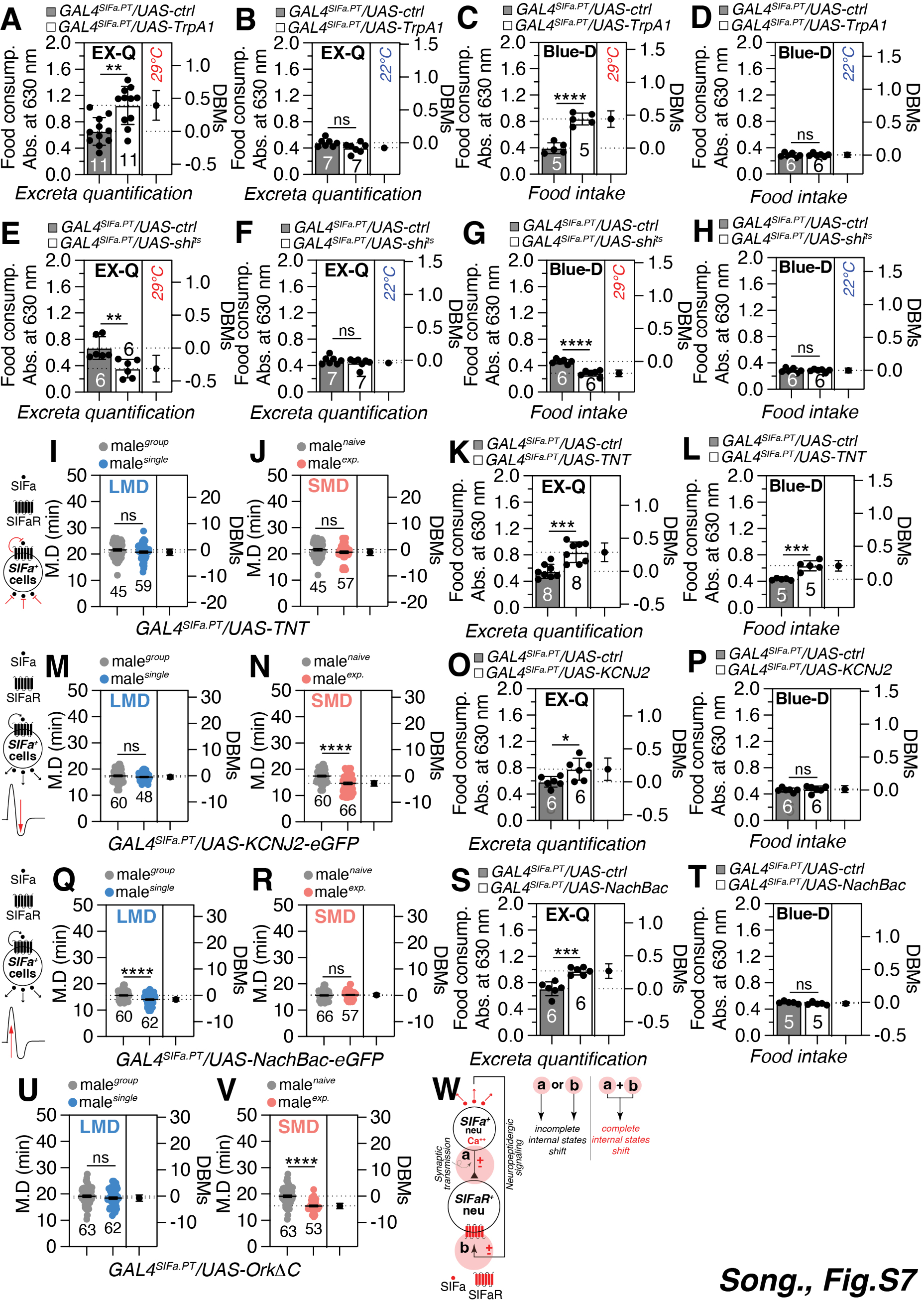

Supplement: S7 Fig — (A–D) 24-h food intake of males measured by EX-Q and Blue-Dye assays of male flies expressing GAL4SIFa.PT driver together with TrpA1 on yeast-sugar medium of different temperatures (A, 29 °C; B, 22 °C; C, 29 °C; D, 22 °C) (two-tailed unpaired t test). In all plots and statistical tests. Data are presented as mean ± s.e.m. ns = not significant (p > 0.05), *p < 0.05, **p < 0.01, ***p < 0.001, ****p < 0.0001. Sample sizes (n) are indicated in the figure panels. See the Materials and methods for a detailed description of the EX-Q and Blue-Dye assay used in this study. (E–H) Blue-Dye and EX-Q assay of flies expressing GAL4SIFa.PT driver together with UAS-shits in 22 and 29 °C (two-tailed unpaired t test). In all plots and statistical tests. Data are presented as mean ± s.e.m. ns = not significant (p > 0.05), *p < 0.05, **p < 0.01, ***p < 0.001, ****p < 0.0001. Sample sizes (n) are indicated in the figure panels. (I, J) MD assays for GAL4SIFa.PT meditated the inactivation of synaptic transmission of SIFa neurons using UAS-TNT (two-tailed unpaired t test). In all plots and statistical tests. Data are presented as mean ± s.e.m. ns = not significant (p > 0.05), *p < 0.05, **p < 0.01, ***p < 0.001, ****p < 0.0001. Sample sizes (n) are indicated in the figure panels. (M-N, Q-R, U-V) MD assays of UAS-KCNJ2-eGFP (M-N), UAS-NachBac-eGFP (Q-R), and UAS-OrkΔC (U-V) crossed with GAL4SIFa.PT (two-tailed unpaired t test). In all plots and statistical tests. Data are presented as mean ± s.e.m. ns = not significant (p > 0.05), *p < 0.05, **p < 0.01, ***p < 0.001, ****p < 0.0001. Sample sizes (n) are indicated in the figure panels. (K-L, O-P, S-T) 24-h excreta quantification of males expressing GAL4SIFa.PT driver together with UAS-TNT (K), UAS-KCNJ2 (M), and UAS-NachBac (Q) on yeast-sugar medium. Blue-Dye assay of male flies expressing GAL4SIFa.PT driver together with UAS-TNT (L), UAS-KCNJ2 (P), and UAS-NachBac (T) (two-tailed unpaired t test). In all plots and statistical tests. Data ar [file pbio.3003345.s008.tif]

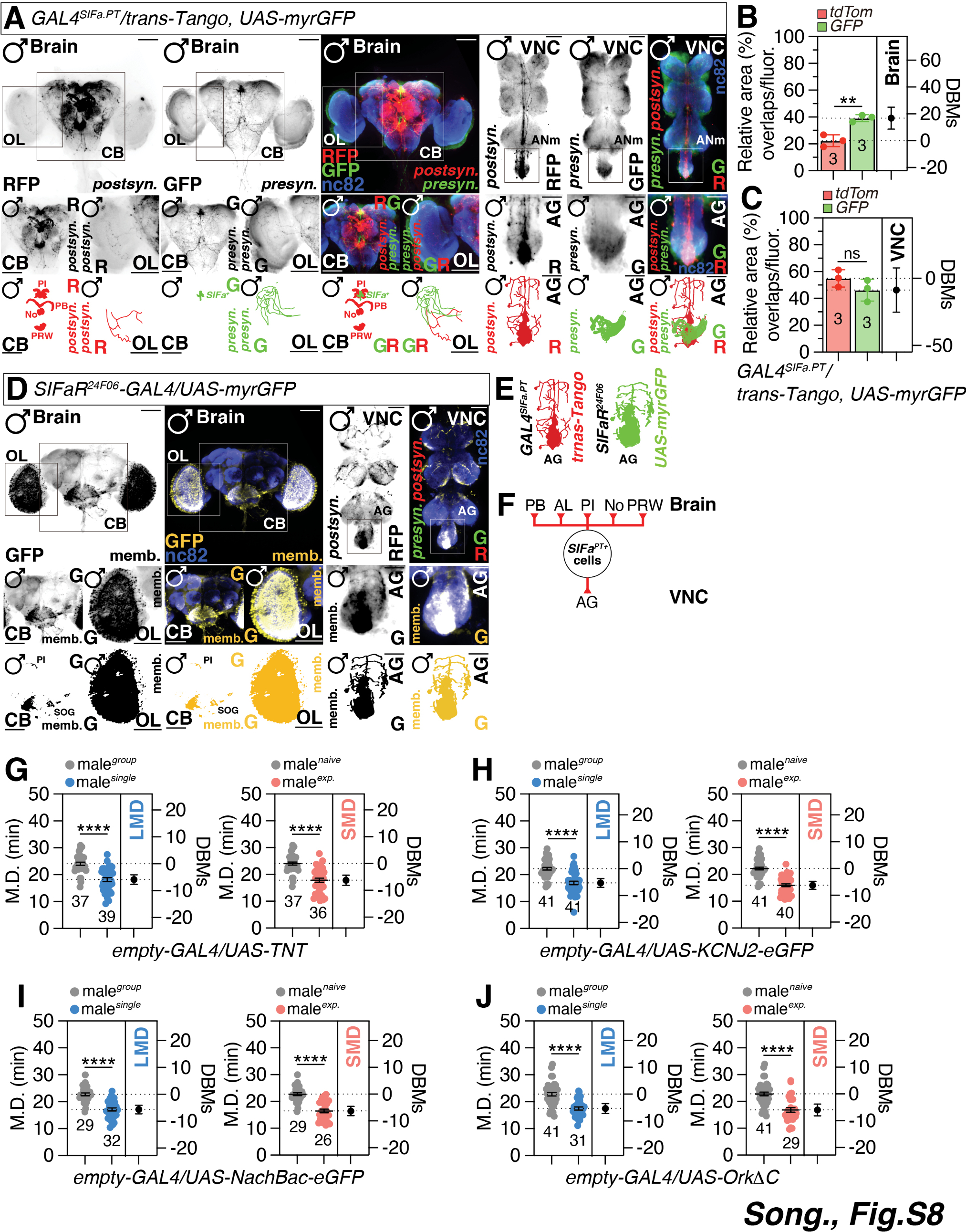

Supplement: S8 Fig — (A) Confocal image of male brain and VNC in which GAL4SIFa.PT was used to simultaneously drive expression of trans-Tango(magenta), visualized with RFP antibody staining, which identifies postsynaptic cells, and myrGFP (green), which labels the GAL4SIFa.PT cells. Areas outlined by boxes in top panels are enlarged in middle. The bottom panels are presented as a red and green scale to show the threshold of RFP and GFP signals marked by threshold function of ImageJ. Scale bars represent 100 μm. (B, C) Quantification of GFP and RFP fluorescence in male fly brain and VNC that expressing GAL4SIFa.PT together with UAS-myrGFP and UAS-trans-Tango (two-tailed unpaired t test). In all plots and statistical tests. Data are presented as mean ± s.e.m. ns = not significant (p > 0.05), *p < 0.05, **p < 0.01, ***p < 0.001, ****p < 0.0001. Sample sizes (n) are indicated in the figure panels. (D) Male flies expressing SIFaR24F06-GAL4 drivers together with UAS-myrGFP were imaged live under a fluorescent microscope with anti-GFP (yellow), and nc82 (blue) antibodies. Areas outlined by boxes are enlarged in the bottom panel. The bottom panels are presented as a yellow scale to show the threshold of GFP signals marked by threshold function of ImageJ. Scale bars represent 100 μm in brain and CB, 10 μm in OL. (E) Comparative examination of AG region SIFa trans-Tango fluorescence signals and SIFaR expression. (F) Schematic illustration of SIFa neurons projecting to PB, AL, AI, PRW, AG, etc. to regulate different behaviors. (G–J) MD assays for GAL4-mediated expression of UAS-TNT (G), UAS-KCNJ2-eGFP (H), UAS-NachBac-eGFP (I), and UAS-OrkΔC using empty-GAL4 driver (two-tailed unpaired t test). In all plots and statistical tests. Data are presented as mean ± s.e.m. ns = not significant (p > 0.05), *p < 0.05, **p < 0.01, ***p < 0.001, ****p < 0.0001. Sample sizes (n) are indicated in the figure panels. Underlying data for all graphs can be found in file S1 Data. (TIF) [file pbio.3003345.s009.tif]

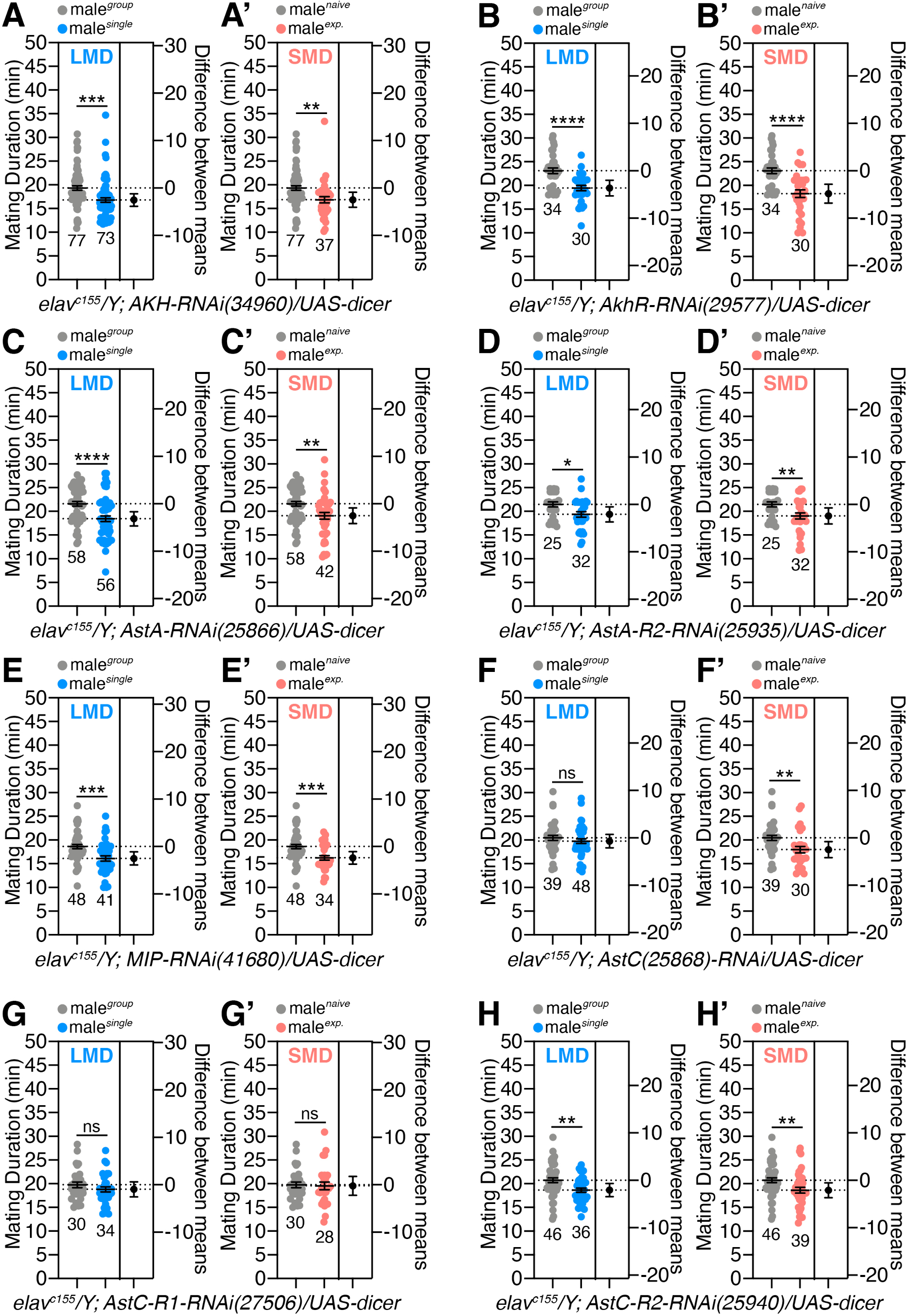

Supplement: S1 File — Fig 1. (A–H′) MD assays for GAL4-mediated knockdown of AKH, AkhR, AstA, AstA-R2, MIP, AstC, AstC-R1, AstC-R2 (BL25940) (in the order of A–H) using the elavc155 driver (two-tailed unpaired t test). In all plots and statistical tests. Data are presented as mean ± s.e.m. ns = not significant (p > 0.05), *p < 0.05, **p < 0.01, ***p < 0.001, ****p < 0.0001. Sample sizes (n) are indicated in the figure panels. (TIF) [file pbio.3003345.s010.tif]

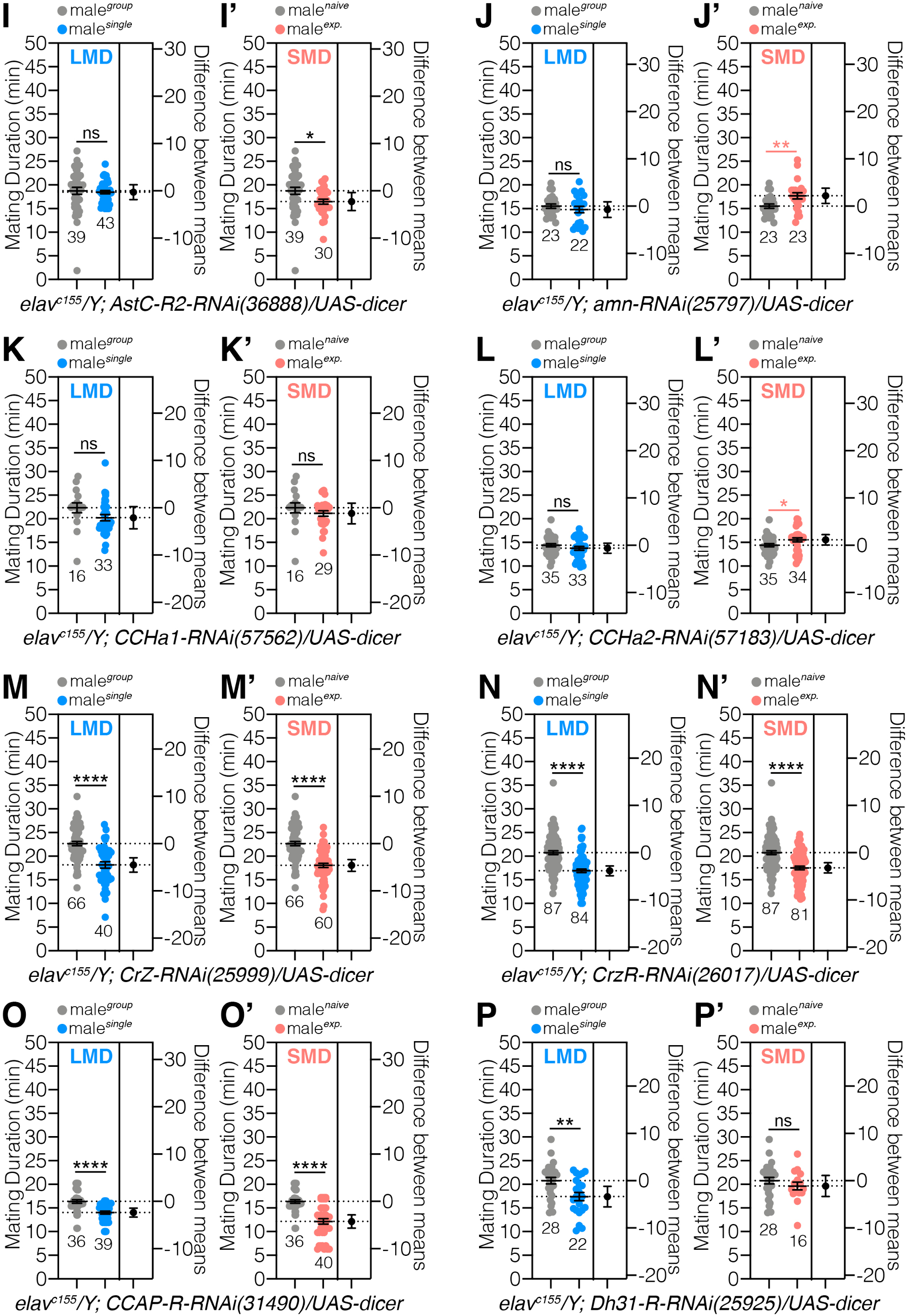

Supplement: S2 File — Fig 1. (I–P′) MD assays for GAL4-mediated knockdown of AstC-R2 (BL36888), amn, CCHa1, CCHa1, CrZ, CrzR, CCAP-R, DH31-R (in the order of I–P) using the elavc155 driver (two-tailed unpaired t test). In all plots and statistical tests. Data are presented as mean ± s.e.m. ns = not significant (p > 0.05), *p < 0.05, **p < 0.01, ***p < 0.001, ****p < 0.0001. Sample sizes (n) are indicated in the figure panels. (TIF) [file pbio.3003345.s011.tif]

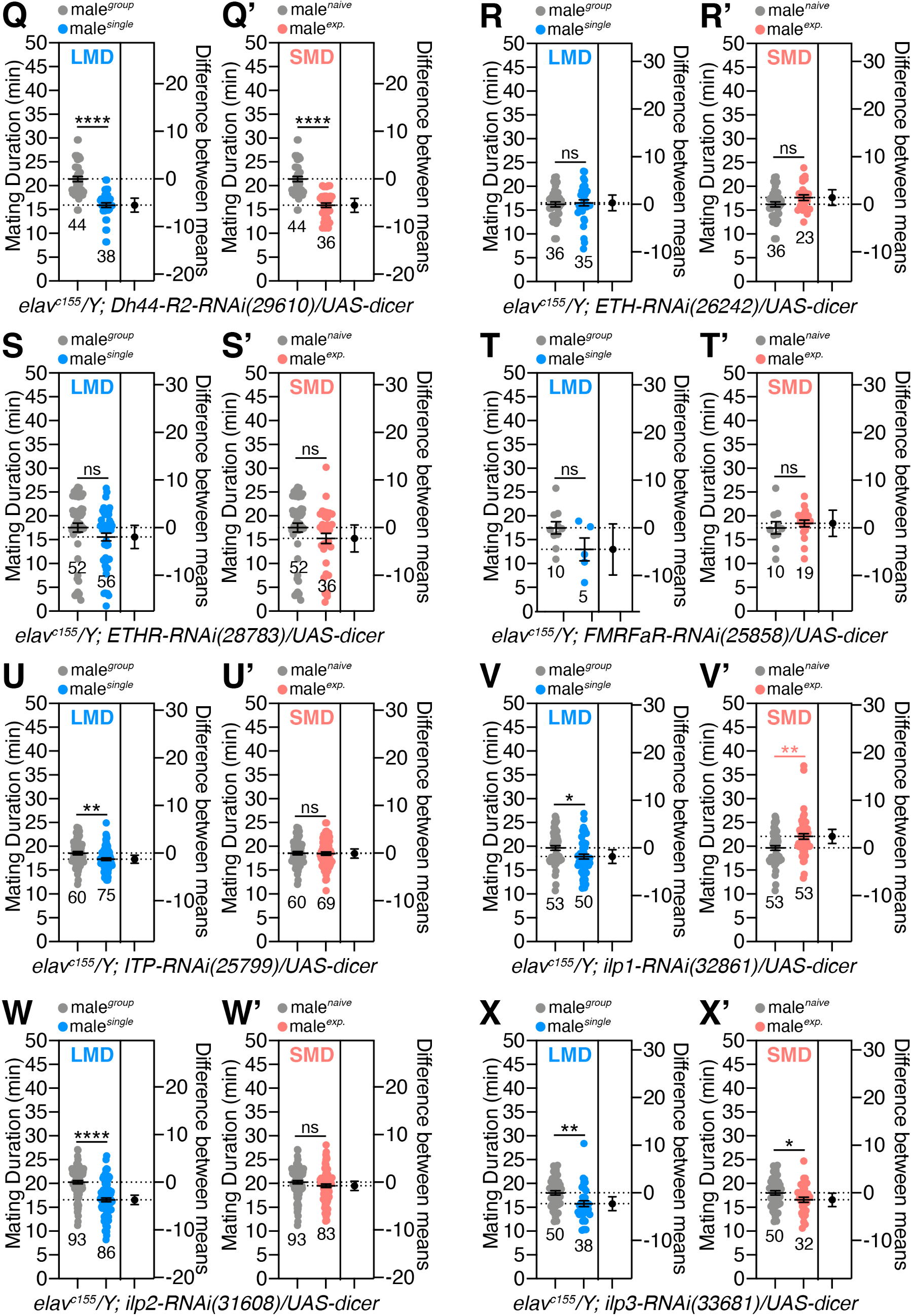

Supplement: S3 File — Fig 1. (Q–X′) MD assays for GAL4 mediated knockdown of Dh44-R2, ETH-RNAi, ETHR, FMRFaR, ITP, ilp1, ilp2, ilp3 (in the order of Q-X) using the elavc155 driver (two-tailed unpaired t test). In all plots and statistical tests. Data are presented as mean ± s.e.m. ns = not significant (p > 0.05), *p < 0.05, **p < 0.01, ***p < 0.001, ****p < 0.0001. Sample sizes (n) are indicated in the figure panels. (TIF) [file pbio.3003345.s012.tif]

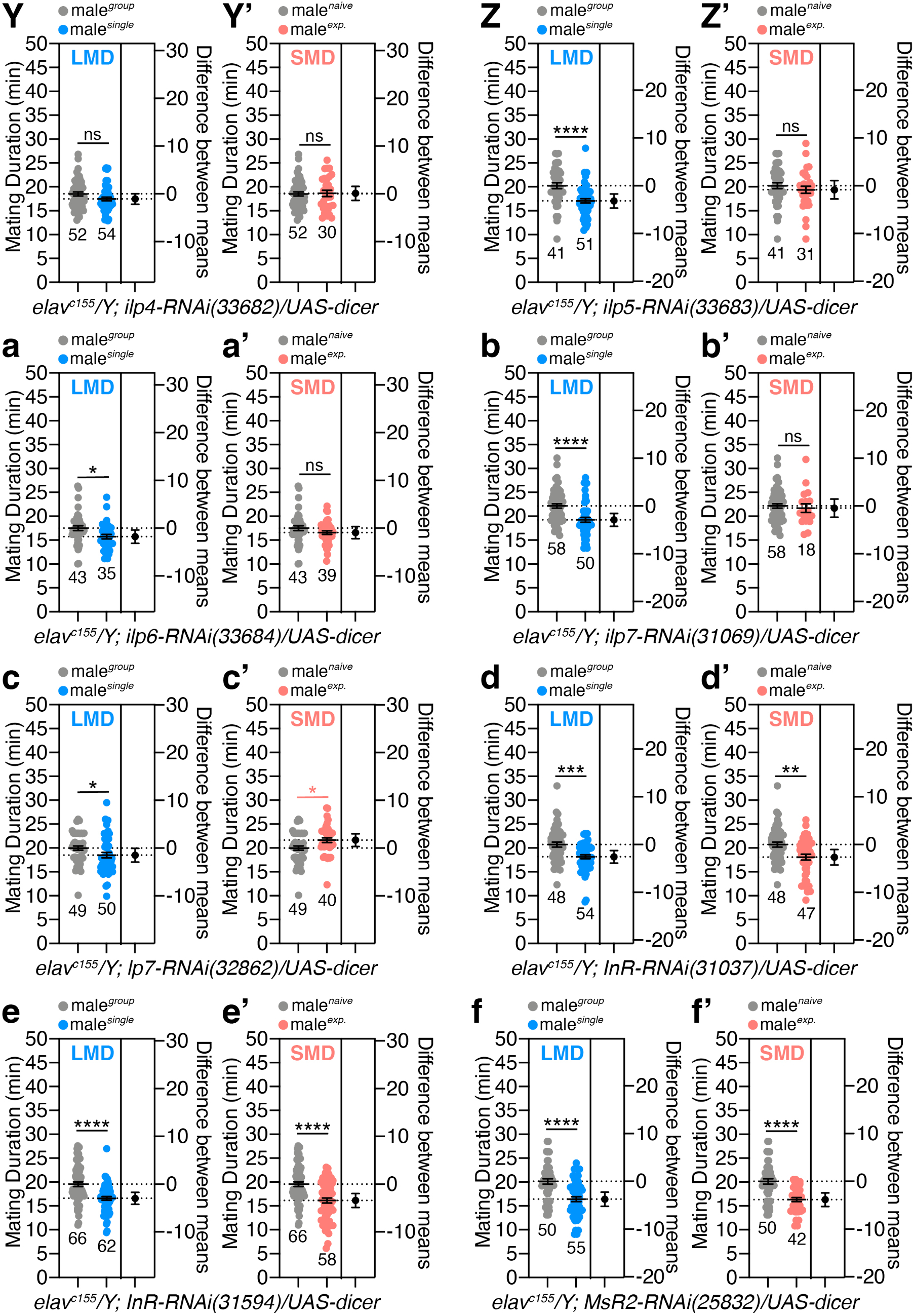

Supplement: S4 File — Fig 1. (Y–F′) MD assays for GAL4-mediated knockdown of ilp4, ilp5, ilp6, ilp7, lp7, InR(31037), InR(31594), MsR2 (in the order of Y–f) using the elavc155 driver (two-tailed unpaired t test). In all plots and statistical tests. Data are presented as mean ± s.e.m. ns = not significant (p > 0.05), *p < 0.05, **p < 0.01, ***p < 0.001, ****p < 0.0001. Sample sizes (n) are indicated in the figure panels. (TIF) [file pbio.3003345.s013.tif]

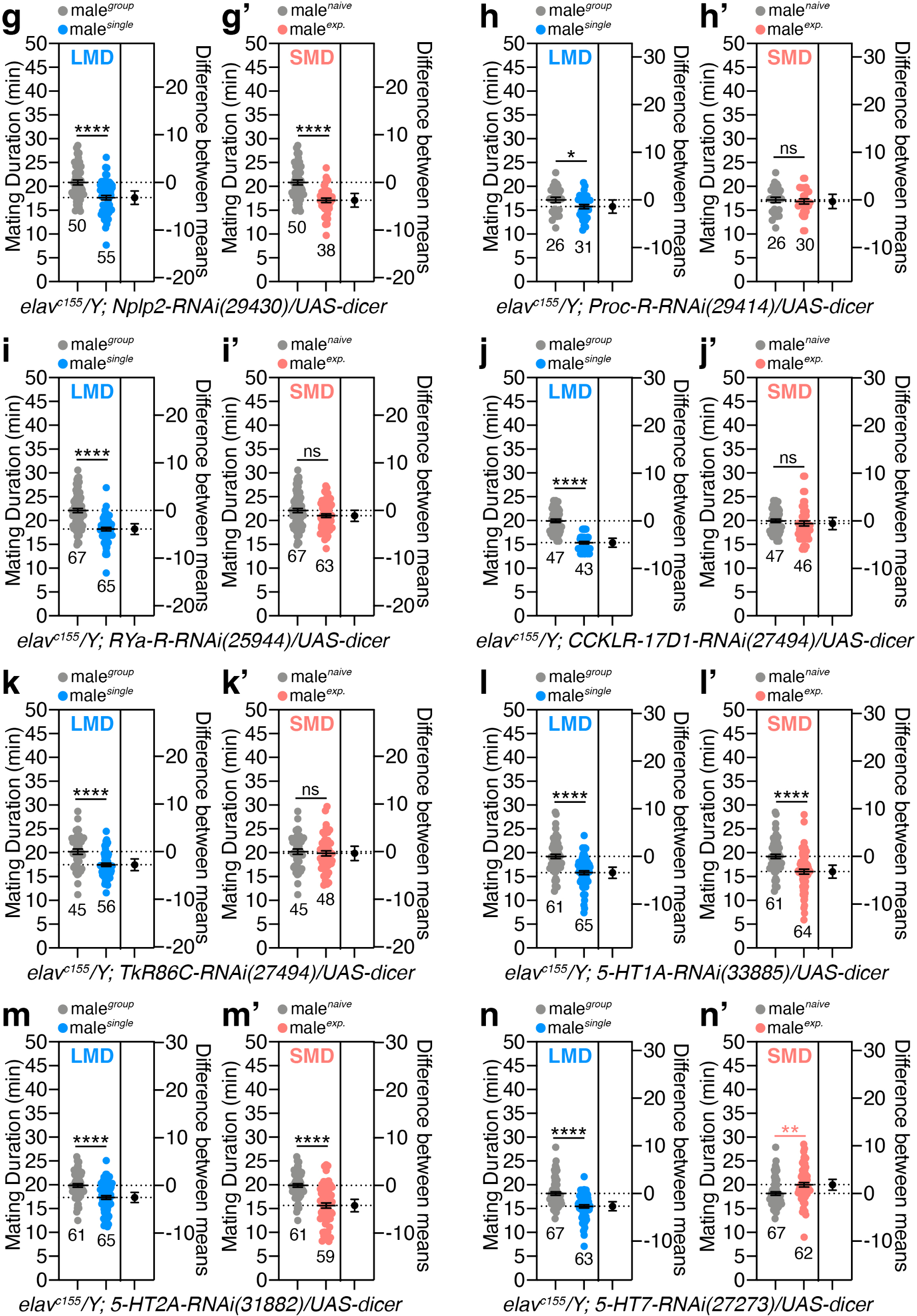

Supplement: S5 File — Fig 1. (G–H′) MD assays for GAL4 mediated knockdown of Nplp2, Proc-R, RYa-R, CCKLR-17D1, TkR86, 5-HT1A, 5-HT2A, 5-HT7 (in the order of g–n) using the elavc155 driver (two-tailed unpaired t test). In all plots and statistical tests. Data are presented as mean ± s.e.m. ns = not significant (p > 0.05), *p < 0.05, **p < 0.01, ***p < 0.001, ****p < 0.0001. Sample sizes (n) are indicated in the figure panels. (TIF) [file pbio.3003345.s014.tif]

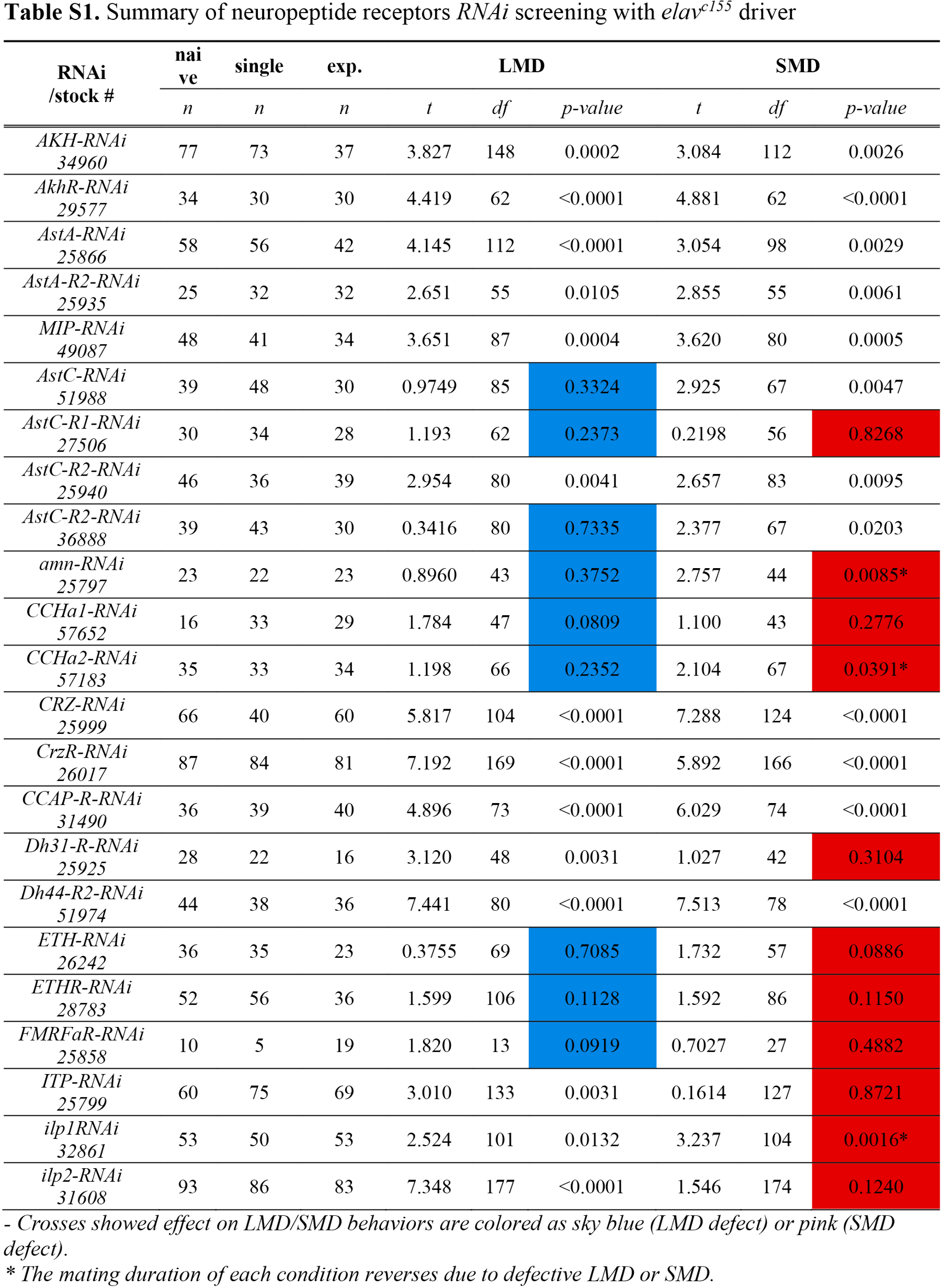

Supplement: S1 Table — (TIF) [file pbio.3003345.s015.tif]
