# Supplementary material for: SIFa peptidergic neurons orchestrate the internal states and energy balance of male Drosophila melanogaster
Source: PLoS Biol. 2025 Sep 4;23(9):e3003345. doi: 10.1371/journal.pbio.3003345 (PMC12410877; doi:10.1371/journal.pbio.3003345)
Supplement: Attachment — Submitted filename: RC_Responce to Reviewer_SIFa_20241203.docx [file pbio.3003345.s017.docx]

**Manuscript number:** RC-2024-02545

**Corresponding author(s):** Woo Jae, Kim

## 1. General Statements

We sincerely appreciate the positive and constructive feedback provided by all three reviewers. Their insightful comments have been invaluable in guiding our revisions. In response, we have made every effort to address their suggestions through additional experiments and by restructuring our manuscript to improve clarity and coherence.

In this revision, we have streamlined the presentation of our data to enhance the narrative flow, ensuring that it is more accessible to a general readership. We believe that these changes not only strengthen our manuscript but also align with the reviewers' recommendations for improvement.

We are hopeful that the revisions we have implemented meet the expectations of the reviewers and contribute to a clearer understanding of our findings. Thank you once again for your thoughtful critiques, which have greatly aided us in refining our work.

## 2. Point-by-point description of the revisions

**Reviewer #1**

***General comment:*** *This manuscript by Song et al. investigates the molecular mechanisms underlying changes in mating duration in Drosophila induced by previous experience. As they have shown previously, they find that male flies reared in isolation have shorter mating duration than those reared in groups, and also that male flies with previous mating experience have shorter mating duration than sexually naïve males. They have conducted a myriad of experiments to demonstrate that the neuropeptide SIFa is required for these changes in mating duration. They have further provided evidence that SIFa-expressing neurons undergo changes in synaptic connectivity and neuronal firing as a result of previous mating experience. Finally, they argue that SIFa neurons form reciprocal connections with sNPF-expressing neurons, and that communication within the SIFa-sNPF circuit is required for experience-dependent changes in mating duration. These results are used to assert that SIFa neurons track the internal state of the flies to modulate behavioral choice.*

**Answer:** We appreciate the reviewer's thoughtful comments and commendations regarding our manuscript. The recognition of our investigation into the molecular mechanisms influencing mating duration in *Drosophila* is greatly valued. In particular, we are grateful for the reviewer's positive remarks about our comprehensive experimental approach to demonstrate the role of the neuropeptide SIFa in these changes. The evidence we provided indicating that SIFa-expressing neurons undergo alterations in synaptic connectivity and neuronal firing due to previous social experiences is crucial for elucidating the underlying neural circuitry involved in experience-dependent behaviors. Finally, we are thankful for the recognition of our assertion that SIFa neurons form reciprocal connections with sNPF-expressing neurons, emphasizing the importance of this circuit in modulating behavioral choices based on internal states. To provide stronger evidence for the interactions between SIFa and sNPF, we conducted detailed GCaMP experiments, which revealed intriguing neural connections between these two neuropeptides. We have included this new data in our main figure. We believe these insights contribute significantly to the existing literature on neuropeptidergic signaling and its implications for understanding complex behaviors in *Drosophila*. We look forward to addressing any further comments and enhancing our manuscript based on your invaluable feedback. Thank you once again for your constructive critique and support.

***Major concerns:***

***Comment 1.*** *The authors are to be commended for the sheer quantity of data they have generated, but I was often overwhelmed by the figures, which try to pack too much into the space provided. As a result, it is often unclear what components belong to each panel. Providing more space between each panel would really help.*

**Answer:** We sincerely appreciate the reviewer’s commendation regarding the extensive data we have generated in our study. It is gratifying to know that our efforts to provide a comprehensive analysis of the molecular mechanisms underlying changes in mating duration have been recognized. We understand the concern regarding the density of information presented in our figures. We aimed to convey a wealth of data to support our findings, but we acknowledge that this may have led to some confusion regarding the organization and clarity of the panels. We are grateful for your constructive feedback on this matter. In response, we have significantly reduced the density of the main figures and decreased the size of the graphs to improve clarity. We have also increased the spacing between panels to ensure that each component is more easily distinguishable. Further details will be provided in our responses to each comment below.

***Comment 2.*** *This is a rare instance where I would recommend paring down the paper to focus on the more novel, clear and relevant results. For example, all of Figure 2 shows the* *projection pattern of SIFa+ neuron dendrites and axons, which have been reported by multiple previous papers. Figure 7G and J show trans-tango data and SIFaR-GAL4 expression patterns, which were previously reported by* *Dreyer et al., 2019. These parts could be removed to supplemental figures. Figure 5 details experiments that knock down expression of different neurotransmitter receptors within the SIFa-expressing cells. The results here are less definitive than the SIFa knockdown results, and the SCope data supporting the idea that these receptors are expressed in SIFa-expressing neurons is equivocal. I would recommend removing these data (perhaps they could serve as the basis for another manuscript) or focusing solely on the CCHa1R results, which is the only manipulation that affects both LMD and SMD.*

**Answer:** We sincerely appreciate the reviewer’s positive feedback regarding the extensive data generated in our study. We also fully agree with the reviewer that the sheer volume of our data made it challenging to support our hypothesis that SIFa neurons serve as a hub for integrating multiple neuropeptide inputs and orchestrating various behaviors related to energy balance, as highlighted in our new Figure 5N.

In response to the reviewer's suggestions, we have streamlined our manuscript by removing excessive and redundant data to enhance clarity and simplicity. First, we have moved Figure 2 to the supplementary materials as the reviewer noted that the branching patterns of SIFa neurons are well-documented in previous literature. Second, we relocated the trans-tango data from Figure 7G to Figure S7, since this information is also well-established. We retained this data in the supplementary section to illustrate the connection of SIFa to our recent findings regarding SIFaR^24F06^ neuron connections. Additionally, we have completely removed the neuropeptide receptor input screening data previously included in Figure 5, as well as Figure S8, which presented fly SCope tSNE data. As suggested by the reviewer, we plan to utilize these data for a future paper focused on investigating the underlying mechanisms of SIFa inputs that modulate SIFa activity. Thanks to the reviewer’s constructive suggestions, we believe our manuscript is now more convincing and clearer for readers.

***Comment 3.*** *Finally, I would like the authors to spend more time explaining how they think the results tie together. For example, how do the authors think the changes in branching and activity in SIFa-expressing neurons tie to the change in mating duration provoked by previous experience? It would benefit the manuscript to simplify and clarify the message about what the authors think is happening at the mechanistic level. The various schematics (eg. Fig 7N) describe the results but the different parts feel like separate findings rather than a single narrative. (MECHANISMS diagram and explanation)*

**Answer:** We appreciate the reviewer’s constructive comments, which have significantly improved our manuscript and conclusions for our readers. As the reviewer will see, we have made substantial revisions in line with the suggestions provided. We dedicated additional time to clarify the electrical activities and synaptic plasticity of SIFa neurons in relation to internal states that orchestrate various behaviors. We have summarized our hypothesis regarding the mechanistic role of SIFa neurons in Figure 5N. In brief, we propose that SIFa neurons function as a hub that receives diverse neuropeptidergic signals, which subsequently alters their electrical activity and synaptic branching. This, in turn, leads to different internal states. The internal states of SIFa neurons can then be interpreted by SIFaR-expressing cells, which help orchestrate various behaviors and physiological responses. We aim to address these aspects further in another manuscript that has been co-submitted alongside this one [1].

***Comment 4.*** Most of the experiments lack traditional controls. For example, in experiments in Fig 1C-K, one would typically include genetic controls that contain either the GAL4 or UAS elements alone. The authors should explain their decision to omit these control experiments and provide an argument for why they are not necessary to correctly interpret the data. In this vein, the authors have stated in the methods that stocks were outcrossed at least 3x to Canton-S background, but 3 outcrosses is insufficient to fully control for genetic background.

**Answer:** We sincerely thank the reviewer for insightful comments regarding the absence of traditional genetic controls in our study of LMD and SMD behaviors. We acknowledge the importance of such controls and wish to clarify our rationale for not including them in the current investigation. The primary reason for not incorporating all genetic control lines is that we have previously assessed the LMD and SMD behaviors of GAL4/+ and UAS/+ strains in our earlier studies. Our past experiences have consistently shown that 100% of the genetic control flies for both GAL4 and UAS exhibit normal LMD and SMD behaviors. Given these findings, we deemed the inclusion of additional genetic controls to be non-essential for the present study, particularly in the context of extensive screening efforts. We understand the value of providing a clear rationale for our methodology choices. To this end, we have added a detailed explanation in the "MATERIALS AND METHODS" section and the figure legends of Figure 1. This clarification aims to assist readers in understanding our decision to omit traditional controls, as outlined below.

"**Mating Duration Assays for Successful Copulation**

The mating duration assay in this study has been reported[33,73,93]. To enhance the efficiency of the mating duration assay, we utilized the Df (1)Exel6234 (DF here after) genetic modified fly line in this study, which harbors a deletion of a specific genomic region that includes the sex peptide receptor (SPR)[94,95]. Previous studies have demonstrated that virgin females of this line exhibit increased receptivity to males[95]. We conducted a comparative analysis between the virgin females of this line and the CS virgin females and found that both groups induced SMD. Consequently, we have elected to employ virgin females from this modified line in all subsequent studies. For naïve males, 40 males from the same strain were placed into a vial with food for 5 days. For single reared males, males of the same strain were collected individually and placed into vials with food for 5 days. For experienced males, 40 males from the same strain were placed into a vial with food for 4 days then 80 DF virgin females were introduced into vials for last 1 day before assay. 40 DF virgin females were collected from bottles and placed into a vial for 5 days. These females provide both sexually experienced partners and mating partners for mating duration assays. At the fifth day after eclosion, males of the appropriate strain and DF virgin females were mildly anaesthetized by CO2. After placing a single female in to the mating chamber, we inserted a transparent film then placed a single male to the other side of the film in each chamber. After allowing for 1 h of recovery in the mating chamber in 25℃ incubators, we removed the transparent film and recorded the mating activities. Only those males that succeeded to mate within 1 h were included for analyses. Initiation and completion of copulation were recorded with an accuracy of 10 sec, and total mating duration was calculated for each couple. All assays were performed from noon to 4pm. Genetic controls with GAL4/+ or UAS/+ lines were omitted from supplementary figures, as prior data confirm their consistent exhibition of normal LMD and SMD behaviors [33,73,93,96,97]. Hence, genetic controls for LMD and SMD behaviors were incorporated exclusively when assessing novel fly strains that had not previously been examined. In essence, internal controls were predominantly employed in the experiments, as LMD and SMD behaviors exhibit enhanced statistical significance when internally controlled. Within the LMD assay, both group and single conditions function reciprocally as internal controls. A significant distinction between the naïve and single conditions implies that the experimental manipulation does not affect LMD. Conversely, the lack of a significant discrepancy suggests that the manipulation does influence LMD. In the context of SMD experiments, the naïve condition (equivalent to the group condition in the LMD assay) and sexually experienced males act as mutual internal controls for one another. A statistically significant divergence between naïve and experienced males indicates that the experimental procedure does not alter SMD. Conversely, the absence of a statistically significant difference suggests that the manipulation does impact SMD. Hence, we incorporated supplementary genetic control experiments solely if they deemed indispensable for testing. All assays were performed from noon to 4 PM. We conducted blinded studies for every test[98,99] .

While we have previously addressed this type of reviewer feedback in our published manuscript [2–7], we appreciate the reviewer’s suggestion to include traditional genetic control experiments. In response, we conducted all feasible combinations of genetic control experiments for LMD/SMD during the revision period. The results are presented in the supplementary figures and are described in the main text.

We appreciate the reviewer's inquiry regarding the genetic background of our experimental lines. In response to the comments, we would like to clarify the following. All of our GAL4, UAS, or RNAi lines, which were utilized as the virgin female stock for outcrosses, have been backcrossed to the Canton-S (CS) genetic background for over ten generations. The majority of these lines, particularly those employed in LMD assays, have been maintained in a CS backcrossed status for several years, ensuring a consistent genetic background across multiple generations. Our experience has indicated that the genetic background, particularly that of the X chromosome inherited from the female parent, plays a pivotal role in the expression of certain behavioral traits. Therefore, we have consistently employed these fully outcrossed females as virgins for conducting experiments related to LMD and SMD behaviors. It is noteworthy that, in contrast to the significance of genetic background for LMD behaviors, we have previously established in our work [6] that the genetic background does not significantly influence SMD behaviors. This distinction is important for the interpretation of our findings. To provide a comprehensive understanding of our experimental design, we have detailed the genetic background considerations in the **"Materials and Methods"** section, specifically in the subsection **"Fly Stocks and Husbandry"** as outlined below.

"To reduce the variation from genetic background, all flies were backcrossed for at least 3 generations to *CS* strain. For the generation of outcrosses, all GAL4, UAS, and RNAi lines employed as the virgin female stock were backcrossed to the *CS* genetic background for a minimum of ten generations. Notably, the majority of these lines, which were utilized for LMD assays, have been maintained in a *CS* backcrossed state for long-term generations subsequent to the initial outcrossing process, exceeding ten backcrosses. Based on our experimental observations, the genetic background of primary significance is that of the X chromosome inherited from the female parent. Consequently, we consistently utilized these fully outcrossed females as virgins for the execution of experiments pertaining to LMD and SMD behaviors. Contrary to the influence on LMD behaviors, we have previously demonstrated that the genetic background exerts negligible influence on SMD behaviors, as reported in our prior publication [6]. All mutants and transgenic lines used here have been described previously."

***Comment 5.*** Throughout the manuscript, the authors appear to use a single control condition (sexually naïve flies raised in groups) to compare to both males raised singly and males with previous sexual experience. These control conditions are duplicated in two separate graphs, one for long mating duration and one for short mating duration, but they are given different names (group vs naïve) depending on the graph. If these are actually the same flies, then this should be made clear, and they should be given a consistent name across the different "experiments".

**Answer:** We are grateful to the reviewer for highlighting the potential for confusion among readers regarding the visualization methods used in our figures. In response to this valuable feedback, we have now included a more detailed explanation of the graph visualization techniques in the legends of Figure 1, as detailed below. This additional information should enhance the clarity and understanding of the figure for all readers.

In the mating duration (MD) assays, light grey data points denote males that were group-reared (or sexually naïve), whereas blue (or pink) data points signify males that were singly reared (or sexually experienced). The dot plots represent the MD of each male fly. The mean value and standard error are labeled within the dot plot (black lines). Asterisks represent significant differences, as revealed by the unpaired Student’s t test, and ns represents non-significant differences M.D represent mating duration. DBMs represent the 'difference between means' for the evaluation of estimation statistics (See **MATERIALS AND METHODS**). Asterisks represent significant differences, as revealed by the Student’s t test (* p<0.05, ** p<0.01, *** p<0.001). Consequently, data points on graphs marked with asterisks indicate that LMD or SMD behaviors remain unaltered or within normal parameters, whereas those labeled with 'ns' signify that LMD or SMD behaviors have been perturbed due to mutations or genetic alterations in the respective strains. For detailed methods, see the **MATERIALS AND METHODS** for a detailed description of the MD assays used in this study. In the framework of our investigation, the routine application of internal controls is employed for the vast majority of experimental procedures, as delineated in the "**Mating Duration Assay**" and "**Statistical Tests**" subsections of the **MATERIALS AND METHODS** section. The same notations for statistical significance are used in other figures.

***Comment 6.*** The authors use SCope data to provide evidence for co-expression of SIFa and other neurotransmitters or neuropeptide receptors. The graphs they show are hard to read and it is not clear to what extent the gene expression is actually overlapping. It would be more definitive to show graphs that indicate which percentage of SIFa-expressing cells co-express other neurotransmitter components, and what the actual level of expression of the genes is. The authors should also provide more information on how they identified the SIFa+ cells in the fly atlas dataset. These are important pieces of information to be able to interpret the effects of manipulation of these other neurotransmitter systems within SIFa-expressing cells on mating duration.

**Answer:** We appreciate the reviewer for pointing out the potential for confusion among readers regarding the visualization methods used in our figures, particularly concerning the tSNE plots of scRNA-seq data. As mentioned in our previous response, we have removed most of the tSNE plots related to co-expression data with SIFa and NPRs, which we believe will reduce any confusion for readers interpreting these plots. However, we have retained a few tSNE plots, specifically Figures 2N-O, to confirm the potential co-expression of the *ple* and *Vglut* genes in SIFa cells. We understand the reviewer’s concerns about the clarity of the presented data and the necessity for more detailed information regarding the extent of co-expression and the identification of SIFa-expressing cells. To address these concerns, we have included a comprehensive description of our methods in the **MATERIALS AND METHODS** section below.

**"Single-nucleus RNA-sequencing analyses**

The snRNAseq dataset analyzed in this paper is published in [112] and available at the Nextflow pipelines (VSN, https://github.com/vib-singlecell-nf), the availability of raw and processed datasets for users to explore, and the development of a crowd-annotation platform with voting, comments, and references through SCope (https://flycellatlas.org/scope), linked to an online analysis platform in ASAP (https://asap.epfl.ch/fca). For the generation of the tSNE plots, we utilized the Fly SCope website (https://scope.aertslab.org/#/FlyCellAtlas/*/welcome). Within the session interface, we selected the appropriate tissues and configured the parameters as follows: 'Log transform' enabled, 'CPM normalize' enabled, 'Expression-based plotting' enabled, 'Show labels' enabled, 'Dissociate viewers' enabled, and both 'Point size' and 'Point alpha level' set to maximum. For all tissues, we referred to the individual tissue sessions within the '10X Cross-tissue' RNAseq dataset. Each tSNE visualization depicts the coexpression patterns of genes, with each color corresponding to the genes listed on the left, right, and bottom of the plot. The tissue name, as referenced on the Fly SCope website is indicated in the upper left corner of the tSNE plot. Dashed lines denote the significant overlap of cell populations annotated by the respective genes. Coexpression between genes or annotated tissues is visually represented by differentially colored cell populations. For instance, yellow cells indicate the coexpression of a gene (or annotated tissue) with red color and another gene (or annotated tissue) with green color. Cyan cells signify coexpression between green and blue, purple cells for red and blue, and white cells for the coexpression of all three colors (red, green, and blue). Consistency in the tSNE plot visualization is preserved across all figures.

Single-cell RNA sequencing (scRNA-seq) data from the Drosophila melanogaster were obtained from the Fly Cell Atlas website (https://doi.org/10.1126/science.abk2432). Oenocytes gene expression analysis employed UMI (Unique Molecular Identifier) data extracted from the 10x VSN oenocyte (Stringent) loom and h5ad file, encompassing a total of 506,660 cells. The Seurat (v4.2.2) package (https://doi.org/10.1016/j.cell.2021.04.048) was utilized for data analysis. Violin plots were generated using the “Vlnplot” function, the cell types are split by FCA.

We have also included detailed descriptions in the figure legends for the initial tSNE plot presented below to help readers clearly understand the significance of this visualization.

"Each tSNE visualization depicts the coexpression patterns of genes, with each color corresponding to the genes listed on the left, right, and/or bottom of the plot. The tissue name, as referenced on the Fly SCope website is indicated in the upper left corner of the tSNE plot. Consistency in the tSNE plot visualization is preserved across all figures."

***Comment 7.*** I would like to see more information on how the thresholding and normalization was done for immunohistochemistry experiments. Was thresholding applied equally across all datasets? Furthermore, "overlap" of Denmark and Syt-eGFP is taken as evidence for synaptic connectivity, but the latter requires more than just overlap in the location of the axon terminal and dendrite regions of the neuron.

**Answer:** Thank you for your continued engagement with our manuscript and for highlighting the need for further clarification on our methods. Your attention to the details of our immunohistochemistry experiments is commendable, and we agree that providing a clear explanation of our thresholding and normalization procedures is essential for the transparency and reproducibility of our results. We concur that the intensity of these signals is indeed correlated with the area measurements, which is a critical factor to consider. In response to the reviewer's valuable suggestion, we have revised our approach and now present our data based on intensity measurements. Additionally, we have updated the labeling of our Y-axis to "Norm. GFP Int.", which stands for "normalized GFP intensity". This change ensures clarity and consistency in the presentation of our data. We primarily adhered to the established methods outlined by Kayser et al. [8]. To address your first point, we have now included a more detailed description of our thresholding and normalization procedures in the **MATERIALS AND METHODS** section as below.

"**Quantitative analysis of fluorescence intensity**

To ascertain calcium levels and synaptic intensity from microscopic images, we dissected and imaged five-day-old flies of various social conditions and genotypes under uniform conditions. The GFP signal in the brains and VNCs was amplified through immunostaining with chicken anti-GFP primary antibody. Image analysis was conducted using ImageJ software. For the quantification of fluorescence intensities, an investigator, blinded to the fly's genotype, thresholded the sum of all pixel intensities within a sub-stack to optimize the signal-to-noise ratio, following established methods [93]. The total fluorescent area or region of interest (ROI) was then quantified using ImageJ, as previously reported. For CaLexA or TRIC signal quantification, we adhered to protocols detailed by Kayser et al. [94], which involve measuring the ROI's GFP-labeled area by summing pixel values across the image stack. This method assumes that changes in the GFP-labeled area and intensity are indicative of alterations in the CaLexA and TRIC signal, reflecting synaptic activity. ROI intensities were background-corrected by measuring and subtracting the fluorescent intensity from a non-specific adjacent area, as per Kayser et al. [94]. For normalization, nc82 fluorescence is utilized for CaLexA, while RFP signal is employed for TRIC experiments, as the RFP signal from the TRIC reporter is independent of calcium signaling [76]. For the analysis of GRASP or tGRASP signals, a sub-stack encompassing all synaptic puncta was thresholded by a genotype-blinded investigator to achieve the optimal signal-to-noise ratio. The fluorescence area or ROI for each region was quantified using ImageJ, employing a similar approach to that used for CaLexA or TRIC quantification [93]. 'Norm. GFP Int.' refers to the normalized GFP intensity relative to the RFP signal."

***Comment 8.*** None of the RNAi experiments have been validated to demonstrate effective knockdown. In many cases, this would be difficult to do because of a lack of an antibody to quantify in a cell-specific manner; however, this fact should be acknowledged, especially in cases where there was found to be a lack of phenotype, which could result from lack of knockdown. The authors could also look for evidence in the literature of cases where RNAi lines they have used have been previously validated. For SIFa, knockdown can be easily confirmed with the SIFa antibody the authors have used elsewhere in the manuscript.
**Answer:** We appreciate the reviewer’s constructive and critical comments regarding the validation of our RNAi experiments through effective knockdown. We understand the reviewer’s concerns about achieving effective knockdown with RNAi; however, we have demonstrated in our unpublished preprint that the neuronal knockdown using independent SIFa-RNAi lines aligns with the SIFa mutant phenotype, which is consistent with our current findings on SIFa knockdown (Wong 2019). In most cases involving RNAi experiments, we have utilized independent RNAi strains to confirm consistent phenotypes and have compared these results with those from mutant phenotypes [1,9]. Therefore, we are confident that our observed SIFa phenotype results from effective RNAi knockdown. Nevertheless, we respect the reviewer’s comments and have conducted additional SIFa knockdown experiments using various GAL4 drivers, followed by immunostaining with SIFa antibodies. As shown in Figure S1B, both neuronal GAL4 drivers and SIFa-GAL4 effectively reduced SIFa immunoreactivity. We believe this indicates that our SIFa knockdown efficiently phenocopies the SIFa mutant phenotype. We also described this result in manuscript as below:

"Using the GAL4^SIFa.PT^ driver and the elav^c155^ driver, we observed a significant decrease in SIFa immunoreactivity following SIFa-RNAi treatment, thereby confirming the effective knockdown of SIFa in these cells. In contrast, when SIFa-RNAi was expressed under the control of the repo-GAL4 driver, no significant change in SIFa immunoreactivity was detected (Fig S1B). This control experiment highlights the specificity of the SIFa-RNAi effect and supports the conclusion that the behavioral changes observed in SMD and LMD are likely attributable to the targeted reduction of SIFa in the intended neuronal populations."

***Minor comments:***

***Comment 1.*** There are quite a lot of citations to preprints, including preprints of the manuscripts under review. It seems inappropriate to cite a preprint of the manuscript you are submitting because it gives a false sense of strengthening the assertions being made in the manuscript.

**Answer:** We agree with the reviewer and have omitted all preprints that are currently under review, except for those that are deemed necessary, such as the Zhang et al. 2024 preprint, which is being submitted alongside this manuscript.

***Comment 2.*** It seems that labels are incorrect on a number of the immunohistochemistry figures. For example, in Fig 2N, it labels dendrites as green, but this is sytEGFP, which is the presynaptic terminal.

**Answer:** We thoroughly reviewed and corrected the errors in the labels.

***Comment 3.*** Fig 4N shows grasp between SIFa-LexA and sNPF-R-GAL4, but the authors have argued that these two components should both be expressed in SIFa-expressing cells. This would make grasp signal misleading, because it would appear in the SIFa-expressing cells even without synaptic contacts due to both split GFP molecules being expressed in these cells.

**Answer:** We appreciate the reviewer’s critical comments regarding the interpretation of our GRASP experiments. As the reviewer noted, we acknowledge that the GRASP results also indicate synaptic contacts between SIFa cells. We have elaborated on these results in the following sections.

"This indicates that the synapses between SIFa cells expressing sNPF-R become stronger (S5K to S5M Fig)."

However, we understand that readers may find the interpretation of this GRASP data confusing, so we have included additional explanations below to clarify.

This indicates that the synapses between SIFa cells expressing sNPF-R become stronger (S5K to S5M Fig) since we have found that SIFa cells express sNPF-R (Fig 3M, S5E and S5G)

***Comment 4.*** For quantifying TRIC and CaLexA experiments (eg. Figure 6A-E), intensity of signal should be measured in addition to the area covered by the signal.

**Answer:** We concur with the reviewer. Since all of our analyses indicated that the area covered by the signal correlates with the signal intensity, we opted to use normalized intensity rather than area coverage.

***Conclusive Comments:*** *This study will be most relevant to researchers interested in understanding neuronal control of behavior. It has provided novel information about the mechanisms underlying mating duration in flies, which is used to delineate how internal state influences behavioral outcomes. This represents a conceptual advance, particularly in identifying a cell type and molecule that influences mating duration decisions. The strength of the manuscript is the number of different assays used to investigate the central question from a number of angles. The limitation is that there is a lack of a big picture tying the different components of the manuscript together. Too much data is presented without providing a framework to understand how the data points fit together.*

**Answer:** We sincerely appreciate the reviewer’s positive feedback regarding our study and the recognition of its relevance to researchers interested in understanding the neuronal control of behavior. We are grateful for the acknowledgment of our novel insights into the mechanisms underlying mating duration in *Drosophila*, particularly in how internal states influence behavioral outcomes. The identification of specific cell types and molecules that affect mating duration decisions indeed represents a significant conceptual advance. We also appreciate the reviewer’s commendation of the diverse array of assays employed in our investigation, which allowed us to approach our central question from multiple perspectives.

In response to the reviewer’s constructive criticism regarding the lack of a cohesive framework tying the various components of our manuscript together, we have completely restructured our manuscript. We removed redundant data and incorporated additional convincing experiments, such as GCaMP analyses, to enhance clarity and coherence. Furthermore, we have provided a simplified yet comprehensive overview that describes the role of SIFa as a hub for neuropeptidergic signaling. This framework illustrates how SIFa orchestrates multiple behaviors related to energy balance through calcium signaling and synaptic plasticity via SIFaR-expressing cells.

We believe these revisions address the reviewer’s concerns and provide a clearer understanding of how the different elements of our study fit together, ultimately strengthening the overall impact of our manuscript. Thank you for your valuable feedback, which has guided us in improving our work.

**Reviewer #2**

***General Comments:*** *In the present study, the authors employ mating behavior in male fruit flies, Drosophila melanogaster, to investigate the behavioral roles of the neuropeptide SIFamide. The duration of mating behavior in these animals varies depending on context, previous experience, and internal metabolic state. The authors use this variability to explore the neuronal mechanisms that control these influences. In an abstraction step, they compare the different mating durations to concepts of neuronal interval timing. 

The behavioral functions of the neuropeptide SIFamide have been thoroughly characterized in several studies, particularly in the contexts of circadian rhythm and sleep, courtship behavior, and food uptake. This study adds new data, demonstrating that SIFamide is essential for the proper control of mating behavior, highlighting the interconnection of various state- and motivation-dependent behaviors at the neuronal level. However, the hypothesis that mating behavior is related to interval timing is not convincingly supported. 

Experimentally, the authors show that RNAi-mediated downregulation of SIFamide affects mating duration in male flies. They use combinations of RNAi lines under the control of various Gal4 lines to identify additional neurotransmitters, neuropeptides, and receptors involved in this process. This approach is complemented by neuroanatomical staining and single-cell RNA sequencing.*

*Overall, the study advances our knowledge about the behavioral roles of SIFamide, which is certainly important, interesting, and worthy of being reported. However, the manuscript also raises several serious caveats and includes points that remain speculative, are less convincing, or are simply incorrect.*

**Answer:** We would like to thank the reviewer for their thoughtful and constructive comments regarding our study. We appreciate the recognition of our investigation into the behavioral roles of the neuropeptide SIFamide in male *Drosophila melanogaster*, particularly how we explored the variability in mating duration influenced by context, previous experience, and internal metabolic state. We are grateful for the acknowledgment that our study adds valuable data demonstrating the essential role of SIFamide in regulating mating behavior, highlighting the interconnectedness of various state- and motivation-dependent behaviors at the neuronal level.

We also appreciate the reviewer's recognition of our experimental approach, which includes RNAi-mediated downregulation of SIFamide, the use of various Gal4 lines to identify additional neurotransmitters, neuropeptides, and receptors involved in this process, as well as our incorporation of neuroanatomical staining and single-cell RNA sequencing.

In response to the reviewer’s concerns regarding the hypothesis that mating behavior is related to interval timing, we acknowledge that this aspect requires further clarification and support. We have revisited this hypothesis in our manuscript to strengthen its foundation and address any speculative elements. We aim to provide more robust evidence and clearer connections between mating behavior and neuronal interval timing.

Furthermore, we have taken care to address any points that may have been perceived as less convincing or incorrect. We are committed to refining our manuscript to ensure that all claims are well-supported by our data. Thank you once again for your valuable feedback. We believe that these revisions will enhance the clarity and impact of our study while addressing the concerns raised.

***Major concerns:***

***Comment 1.*** *The authors conclude from their mating experiments that SIFamide controls interval timing. This conclusion is not supported by the data, which only indicate that SIFamide is required for normal mating duration and modulates the motivation-dependent component of this behavior. There is no clear evidence linking this to interval timing.*

**Answer:** We appreciate the reviewer’s insightful comments regarding our conclusion linking SIFamide to interval timing in mating behavior. We acknowledge that our data primarily demonstrate that SIFamide is required for normal mating duration and modulates the motivation-dependent aspects of this behavior, and we recognize the need for clearer evidence connecting these observations to interval timing. Current research by Crickmore et al. has shed light on how mating duration in *Drosophila* serves as a powerful model for exploring changes in motivation over time as behavioral goals are achieved. For instance, at approximately six minutes into mating, sperm transfer occurs, leading to a significant shift in the male's nervous system: he no longer prioritizes sustaining the mating at the expense of his own survival. This change is driven by the output of four male-specific neurons that produce the neuropeptide Corazonin (Crz). When these Crz neurons are inhibited, sperm transfer does not occur, and the male fails to downregulate his motivation, resulting in matings that can last for hours instead of the typical ~23 minutes [10].

Recent research by Crickmore et al. has received NIH R01 funding (Mechanisms of Interval Timing, 1R01GM134222-01) to explore mating duration in *Drosophila* as a genetic model for interval timing. Their work highlights how changes in motivation over time can influence mating behavior, particularly noting that significant behavioral shifts occur during mating, such as the transfer of sperm at approximately six minutes, which correlates with a decrease in the male's motivation to continue mating [10]. These findings suggest that mating duration is not only a behavioral endpoint but may also reflect underlying mechanisms related to interval timing.

We believe that by leveraging the robustness and experimental tractability of these findings, along with our own work on SIFamide's role in mating behavior, we can gain deeper insights into the molecular and circuit mechanisms underlying interval timing. We will revise our manuscript to clarify this relationship and emphasize how SIFamide may interact with other neuropeptides and neuronal circuits involved in motivation and timing.

In addition to the efforts of Crickmore's group to connect mating duration with a straightforward genetic model for interval timing, we have previously published several papers demonstrating that LMD and SMD can serve as effective genetic models for interval timing within the fly research community. For instance, we have successfully connected SMD to an interval timing model in a recently published paper [6], as detailed below:

"We hypothesize that SMD can serve as a straightforward genetic model system through which we can investigate "interval timing," the capacity of animals to distinguish between periods ranging from minutes to hours in duration.....

In summary, we report a novel sensory pathway that controls mating investment related to sexual experiences in *Drosophila*. Since both LMD and SMD behaviors are involved in controlling male investment by varying the interval of mating, these two behavioral paradigms will provide a new avenue to study how the brain computes the ‘interval timing’ that allows an animal to subjectively experience the passage of physical time [11–16]."

Lee, S. G., Sun, D., Miao, H., Wu, Z., Kang, C., Saad, B., ... & Kim, W. J. (2023). Taste and pheromonal inputs govern the regulation of time investment for mating by sexual experience in male Drosophila melanogaster. *PLoS Genetics*, *19*(5), e1010753.

We have also successfully linked LMD behavior to an interval timing model and have published several papers on this topic recently [4,5,7].

Sun, Y., Zhang, X., Wu, Z., Li, W., & Kim, W. J. (2024). Genetic Screening Reveals Cone Cell-Specific Factors as Common Genetic Targets Modulating Rival-Induced Prolonged Mating in male Drosophila melanogaster. *G3: Genes, Genomes, Genetics*, jkae255.

Zhang, T., Zhang, X., Sun, D., & Kim, W. J. (2024). Exploring the Asymmetric Body’s Influence on Interval Timing Behaviors of Drosophila melanogaster. *Behavior Genetics*, *54*(5), 416-425.

Huang, Y., Kwan, A., & Kim, W. J. (2024). Y chromosome genes interplay with interval timing in regulating mating duration of male Drosophila melanogaster. *Gene Reports*, *36*, 101999.

Finally, in this context, we have outlined in our INTRODUCTION section below how our LMD and SMD models are related to interval timing, aiming to persuade readers of their relevance. We hope that the reviewer and readers are convinced that mating duration and its associated motivational changes such as LMD and SMD provide a compelling model for studying the genetic basis of interval timing in *Drosophila*.

"The mating duration of male fruit flies is a suitable model for studying interval timing and it could change based on internal states and environmental context. Previous studies by our group[27–30] and others[31,32] have established several frameworks for investigating the mating duration using sophisticated genetic techniques that can analyze and uncover the neural circuits’ principles governing interval timing. In particular, males exhibit LMD behavior when they are exposed to an environment with rivals, which means they prolong their mating duration. Conversely, they display SMD behavior when they are in a sexually saturated condition, meaning they reduce their mating duration[33,34]."

***Comment 2.*** On line 160, the authors state, "The connection between the dendrites and axons of the SIFamide neuronal processes is unknown." This is not entirely correct. State-of-the-art connectome analyses can determine synaptic connectivities between SIFamidergic neurons and pre-/postsynaptic neurons. The authors also overlook the thorough connectivity analysis by Martelli et al. (2017), which includes functional analyses and detailed anatomical descriptions that the current study confirms.

**Answer:** We appreciate the reviewer for acknowledging the efforts of Martelli et al. in elucidating the neuronal architecture of SIFa neurons. We recognize that it was an oversight on our part to state that "the connection between the dendrites and axons of SIFa neurons is unknown." This error arose because our manuscript has been in preparation for over ten years, predating the publication of Martelli et al.'s work. That statement likely reflects an outdated section of the manuscript.

We fully acknowledge the findings from previous publications and have removed that sentence entirely from our manuscript. In its place, we have added the following statement:

"The established connections and architecture of SIFa neurons has been described by Martelli et al., which enhances our understanding of their functional roles within the neuronal circuitry [51]. To identify the dendritic and axonal components of SIFa-neuronal processes, we employed a similar approach to that reported by Martelli [51]."

Thank you for your valuable feedback, which has helped us improve the clarity and accuracy of our manuscript.

***Comment 3.*** *The mating experiments are overall okay, with sufficiently high sample sizes and appropriate statistical tests. However, many experiments lack genetic controls for the heterozygous parental strains, such as Gal4-ines AND UAS-lines. This is of course of importance and common standard.*

**Answer:** While we have previously addressed this type of reviewer feedback in our published manuscript [2–7] as well as this manuscript by Reviewer #1, we appreciate the reviewer’s suggestion to include traditional genetic control experiments. In response, we conducted all feasible combinations of genetic control experiments for LMD/SMD during the revision period. The results are presented in the supplementary figures and are described in the main text.

***Comment 4.*** *Using a battery of RNAi lines, the authors aim to uncover which neurotransmitters might be co-released from SIFamide neurons to influence mating behavior. However, a behavioral effect of an RNAi construct expressed in SIFamidergic neurons does not demonstrate that the respective transmitter is actually released from these neurons. Alternative methods are needed to show whether glutamate, dopamine, serotonin, octopamine, etc., are present and released from SIFamide neurons. It is particularly challenging to prove that a certain substance acts as a transmitter released by a specific neuron. For example, anti-Tdc2 staining does not actually cover SIFamide neurons, and dopamine has not been described as present in SIFamide neurons.*

**Answer:** We appreciate the reviewer’s constructive comments regarding the need to demonstrate the presence of the responsible neurotransmitters in SIFa neurons. While many studies utilize neurotransmitter-synthesizing enzymes such as TH, VGlut, Gad1, and Trhn to assess neurotransmitter effects, we recognize the importance of conclusively establishing that glutamate and dopamine play significant roles in modulating energy balance within SIFa neurons.

First, the enrichment of tyramine (TA), octopamine (OA), and dopamine (DA) in SIFa neurons was suggested in the study by Croset et al. (2018) [17]. Although we tested Tdc2-RNAi and observed interesting phenotypes, we chose not to publish these findings, as our data on glutamate and dopamine provide a more compelling explanation for how SIFa cotransmission with these neurotransmitters can independently influence various behaviors, including sleep and mating duration.

To confirm the expression of DA in SIFa neurons, we employed a well-established genetic toolkit for dissecting dopamine circuit function in *Drosophila* [18]. Our findings indicate that TH-C-GAL4 specifically labels SIFa neurons, which have been confirmed as dopaminergic (S4M Fig). Our genetic intersection data, along with Xie et al.'s findings from 2018, confirm that a subset of SIFa neurons is indeed dopaminergic. We have described these new results in the main text as follows:

To further verify the presence of DA neurons within the SIFa neuron population, we utilized a well-established genetic toolkit for dissecting DA circuits and confirmed part of SIFa neurons are dopaminergic (S4M Fig) [58].

To confirm the glutamatergic characteristics of SIFa neurons, we conducted several experiments that established glutamate as the most critical neurotransmitter for generating interval timing in both SIFa and SIFaR neurons. First, to demonstrate the presence of glutamatergic synaptic vesicles in SIFa neurons, we utilized a conditional glutamatergic synaptic vesicle marker for *Drosophila*, developed by Certel et al. [19]. Our results confirmed that SIFa neurons exhibit strong expression of glutamatergic synaptic vesicles (Fig. 2P and Fig. S4N as a genetic control). We have described these new results in the main text as follows:

“To further verify the presence of DA neurons within the SIFa neuron population, we utilized a well-established genetic toolkit for dissecting DA circuits and confirmed part of SIFa neurons are dopaminergic (S4M Fig) [58]. We also employed a conditional glutamatergic synaptic vesicle marker to confirm the presence of glutamatergic SIFa neurons (Fig 2P and Fig S4N) [59].”

To further confirm that glutamate release from SIFa neurons influences the function of SIFaR neurons, we tested several RNAi strains targeting glutamate receptors. Our results showed that the knockdown of glutamate receptors in SIFaR-expressing neurons produced phenotypes similar to those observed with VGlut-RNAi knockdown in SIFa neurons (S4G-L Fig). We believe that this series of experiments demonstrates that glutamate and dopamine work in conjunction with SIFa to modulate interval timing and other behaviors related to energy balance. We have described these new results in the main text as follows:

"To further substantiate the role of glutamate in SIFa-mediated behaviors. we targeted knockdown of VGlut receptors in SIFaR-expressing neurons. Strikingly, the knockdown of VGlut receptors in these neurons also disrupted SMD behavior, mirroring the phenotype observed upon direct suppression of glutamatergic signaling in SIFa neurons (S4G-L Fig). This suggests that glutamate is an essential neurotransmitter for modulating interval timing in SIFa neurons.”

***Comment 5.*** Single-cell RNA sequencing data alone is insufficient to claim multiple transmitter co-release from SIFamide neurons. Figures illustrating single-cell RNA sequencing, such as Figure 3P-R, are not intuitively understandable, and the figure legends lack sufficient information to clarify these panels. As a side note, Tdc2 is not only present in octopaminergic neurons, but also in tyraminergic neurons.

**Answer:** We agree with the reviewer that scRNA-seq data alone is insufficient to support claims of multiple transmitter co-release in SIFa neurons. We also appreciate the reviewer for highlighting the potential for confusion among readers regarding the visualization methods used in our figures, particularly the tSNE plots of the scRNA-seq data. As noted in our previous response to Reviewer #1, we have removed most of the tSNE plots related to co-expression data involving SIFa and NPRs, which we believe will help clarify the interpretation for readers. However, we have retained a few tSNE plots, specifically Figures 2N-O, to illustrate the potential co-expression of the ple and Vglut genes in SIFa cells.

We understand the reviewer’s concerns regarding the clarity of the presented data and the need for more detailed information about the extent of co-expression and the identification of SIFa-expressing cells. To address these concerns, we have provided a comprehensive description of our methods in the **MATERIALS AND METHODS** section below.

**"Single-nucleus RNA-sequencing analyses**

The snRNAseq dataset analyzed in this paper is published in [20]and available at the Nextflow pipelines (VSN, https://github.com/vib-singlecell-nf), the availability of raw and processed datasets for users to explore, and the development of a crowd-annotation platform with voting, comments, and references through SCope (https://flycellatlas.org/scope), linked to an online analysis platform in ASAP (https://asap.epfl.ch/fca). For the generation of the tSNE plots, we utilized the Fly SCope website (https://scope.aertslab.org/#/FlyCellAtlas/*/welcome). Within the session interface, we selected the appropriate tissues and configured the parameters as follows: 'Log transform' enabled, 'CPM normalize' enabled, 'Expression-based plotting' enabled, 'Show labels' enabled, 'Dissociate viewers' enabled, and both 'Point size' and 'Point alpha level' set to maximum. For all tissues, we referred to the individual tissue sessions within the '10X Cross-tissue' RNAseq dataset. Each tSNE visualization depicts the coexpression patterns of genes, with each color corresponding to the genes listed on the left, right, and bottom of the plot. The tissue name, as referenced on the Fly SCope website is indicated in the upper left corner of the tSNE plot. Dashed lines denote the significant overlap of cell populations annotated by the respective genes. Coexpression between genes or annotated tissues is visually represented by differentially colored cell populations. For instance, yellow cells indicate the coexpression of a gene (or annotated tissue) with red color and another gene (or annotated tissue) with green color. Cyan cells signify coexpression between green and blue, purple cells for red and blue, and white cells for the coexpression of all three colors (red, green, and blue). Consistency in the tSNE plot visualization is preserved across all figures.

Single-cell RNA sequencing (scRNA-seq) data from the *Drosophila melanogaster* were obtained from the Fly Cell Atlas website (https://doi.org/10.1126/science.abk2432). Oenocytes gene expression analysis employed UMI (Unique Molecular Identifier) data extracted from the 10x VSN oenocyte (Stringent) loom and h5ad file, encompassing a total of 506,660 cells. The Seurat (v4.2.2) package (https://doi.org/10.1016/j.cell.2021.04.048) was utilized for data analysis. Violin plots were generated using the “Vlnplot” function, the cell types are split by FCA."

We have also included detailed descriptions in the figure legends for the initial tSNE plot presented below to help readers clearly understand the significance of this visualization.

"Each tSNE visualization depicts the coexpression patterns of genes, with each color corresponding to the genes listed on the left, right, and/or bottom of the plot. The tissue name, as referenced on the Fly SCope website is indicated in the upper left corner of the tSNE plot. Consistency in the tSNE plot visualization is preserved across all figures."

We appreciate the reviewer for acknowledging that Tdc2 is present in both TA and OA neurons. As we mentioned earlier, we have completely removed the Tdc2-related results from this manuscript, as we believe that more detailed experiments are necessary to confirm the roles of TA and OA in SIFa neurons.

***Comment 6.*** *The same argument applies to the expression of sNPF receptors in SIFamide neurons. The rather small anatomical stainings shown in figure 4M do not convincingly and unambiguously show that actually sNPF receptors are located on SIFamide neurons.*

**Answer:** We appreciate the reviewer for pointing out that the co-expression of sNPF-R and SIFa needs further verification, and we agree with this assessment. To confirm the co-expression of SIFa with sNPF-R, we conducted a mini-screen of various sNPF-R driver lines and found that the chemoconnectome (CCT) sNPF-R2A driver which represent the physiological expression patterns of sNPF-R, consistently labels SIFa neurons [21].

To further establish the functional connection between the SIFa and sNPF systems, we performed GCaMP experiments using SIFa-driven GCaMP in conjunction with sNPF-R neurons expressing P2X2, which can be activated by ATP treatment. As shown in Figures 3N-P, we demonstrated that activation of sNPF-R neurons by ATP significantly increases calcium levels in SIFa neurons. Our results strongly suggest that the sNPF-sNPF-R/SIFa system is functionally present and plays a role in modulating interval timing behaviors.

***Comment 7.*** *The authors use the GRASP technique (figure 4N) to determine whether synaptic connections are subject to modulation as a result from the animals' individual experience. The overall extremely bright fluorescence at the dorsal areas of both brain hemispheres (figure 4 N, middle panel) raises doubts whether this signal is actually a specific GRASP fluorescence between two small populations of neurons.*

**Answer:** We appreciate the reviewer for critically highlighting the inadequacies in our presentation of the GRASP data. We agree that one of our previous panels contained excessive background noise, making it difficult for reviewers and readers to discern the different neuronal connections. To address this issue, we have replaced it with a more representative image that clearly illustrates the strengthening of synaptic connections from SIF to sNPF-R in several neurons, including SIFa cells (Fig. S5J). We hope that this updated image will help convince both the reviewer and readers of the validity of our GRASP data.

***Comment 8.*** *The authors cite Martelli et al. (2017) with the hypothesis that sNPF-releasing neurons provide input signals to SIFamide neurons to modulate feeding behavior. However, the cited manuscript does not contain such a hypothesis. The authors should review the reference in more detail.*

**Answer:** We appreciate reviewer to correctly point our misunderstanding of references. We agree with reviewer that Martelli et al.'s paper didn't mention about sNPF signaling transmits hunger and satiety information to SIFa neurons. We removed this sentence and replaced it as below correctly mentioning that sNPF signaling is related to feeding behavior however it's connection to SIFa neurons are not known. We greatly appreciate the reviewer for acknowledging our efforts to accurately cite previous articles that support our rationale and ideas.

" Short neuropeptide F (sNPF) signaling plays a crucial role in regulating feeding behavior in Drosophila melanogaster, influencing food intake and body size [60,66,67]. However, there is currently no direct evidence reported linking sNPF signaling to SIFa neurons."

***Comment 9.*** *In lines 281 ff., the authors state that SIFamide neurons receive inputs from peptidergic neurons but simultaneously claim that "this speculation is based on morphological observations." This is incorrect. The functional co-activation/imaging analyses provided in Martelli et al. (2017) should not be ignored.*
**Answer:** We fully agree with the reviewer that we misinterpreted Martelli et al.'s analysis. We have removed *"this speculation is based on morphological observations." from* the following sentence and finalize as below:

"The SIFa neurons receive inputs from many peptidergic pathways including Crz, dilp2, Dsk, sNPF, MIP, and hugin"

***Comment 10.*** *Figure 6: A transcriptional calcium sensor (TRIC) was used to quantify the accumulation GFP induced by calcium influx in SIFamide neurons. However, I could not find any description of the method in the materials and methods section, nor any explanation how the data were acquired or analyzed. What is the RFP expression good for? How exactly are thresholds determined, and why are areas rather than fluorescence intensities quantified? Overall, this part of the manuscript is rather confusing and needs more explanation.*

**Answer:** Thank you for your continued engagement with our manuscript and for highlighting the need for further clarification on our methods. Your attention to the details of our immunohistochemistry experiments is commendable, and we agree that providing a clear explanation of our thresholding and normalization procedures is essential for the transparency and reproducibility of our results. We primarily adhered to the established methods outlined by Kayser et al. [8]. To address your first point, we have now included a more detailed description of our thresholding and normalization procedures in the **MATERIALS AND METHODS** section as below.

"**Quantitative analysis of fluorescence intensity**

To ascertain calcium levels and synaptic intensity from microscopic images, we dissected and imaged five-day-old flies of various social conditions and genotypes under uniform conditions. The GFP signal in the brains and VNCs was amplified through immunostaining with chicken anti-GFP, rabbit anti-DsRed, and mouse anti-nc82 primary antibodies. Image analysis was conducted using ImageJ software. For the quantification of fluorescence intensities, an investigator, blinded to the fly's genotype, thresholded the sum of all pixel intensities within a sub-stack to optimize the signal-to-noise ratio, following established methods [100]. The total fluorescent area or region of interest (ROI) was then quantified using ImageJ, as previously reported. For CaLexA or TRIC signal quantification, we adhered to protocols detailed by Kayser et al. [101], which involve measuring the ROI's GFP-labeled area by summing pixel values across the image stack. This method assumes that changes in the GFP-labeled area and intensity are indicative of alterations in the CaLexA and TRIC signal, reflecting synaptic activity. ROI intensities were background-corrected by measuring and subtracting the fluorescent intensity from a non-specific adjacent area, as per Kayser et al. [101]. For normalization, nc82 fluorescence is utilized for CaLexA, while RFP signal is employed for TRIC experiments, as the RFP signal from the TRIC reporter is independent of calcium signaling [72] . For the analysis of GRASP or tGRASP signals, a sub-stack encompassing all synaptic puncta was thresholded by a genotype-blinded investigator to achieve the optimal signal-to-noise ratio. The fluorescence area or ROI for each region was quantified using ImageJ, employing a similar approach to that used for CaLexA or TRIC quantification [100]. 'Norm. GFP Int.' refers to the normalized GFP intensity relative to the RFP signal.

***Comment 11.*** Similarly, it remains unclear how exactly syteGFP fluorescence and DenMark fluorescence were quantified. Why are areas indicated and not fluorescence intensity values? In fact, it appears worrisome that isolation of males should lead to a drastic decline in synaptic terminals (as measure through a vesicle-associated protein) by ~ 30%, or, conversely, keeping animals in groups lead to an respective increase (figure 7D). The technical information how exactly this was quantified is not sufficient.

**Answer:** Thank you for your ongoing engagement with our manuscript and for emphasizing the need for clarification on our methods. We appreciate your attention to the details of our immunohistochemistry experiments and agree that a clear explanation of our thresholding and normalization procedures is vital for transparency and reproducibility. We acknowledge that signal intensity correlates with area measurements, which is an important consideration. In response to your valuable suggestion, we have revised our approach to present data based on intensity measurements and updated the Y-axis labeling to "Norm. GFP Int." (normalized GFP intensity) for clarity. We primarily followed the established methods from Kayser et al. (2014) [8]. Additionally, we have included a more detailed description of our thresholding and normalization procedures in the "Quantitative analysis of fluorescence intensity" in **MATERIALS AND METHODS** section as we quoted above.

***Minor concerns:***

***Comment 1.*** Reference 29 and reference 33 are the same.

**Answer:** We removed reference 29.

***Comment 2.*** In figure legends, abbreviations should be explained when used first (e.g., figure 1 A "MD", is explained below for panel C-F), or "CS males".
**Answer:** We have ensured that abbreviations are explained only when they are first used in the figure legends.

***Comment 3.*** Indications for statistical significance must be shown in all figure legends at the end of each figure legend, not only in figure 1.
**Answer:** We appreciate the reviewer’s advice. However, we have published all our other manuscripts using the same format for mating duration, stating, "The same notations for statistical significance are used in other figures," in the first figure where we describe our statistical significances. We intend to continue with this approach initially and will then adhere to the journal's policy.

***Comment 4.*** The figures appear overloaded. For example why do you need two different axis designations (mating duration and differences between means)?
**Answer:** We appreciate the reviewer's suggestion to refine our figures, and we have indeed reformatted them to provide clearer presentation and improved readability. Our decision is based on the fact that our analysis encompasses not only traditional t-tests but also incorporates estimation statistics, which have been demonstrated to be effective for biological data analysis [22]. The inclusion of DBMs is essential for the accurate interpretation of these estimation statistics, ensuring a comprehensive representation of our findings. This is the primary area where we present two different axis designations.

***Comment 5.*** Line: 1154: Typo: gluttaminergic should be glutamatergic.

**Answer:** We fixed all.

***Comment 6.*** The authors frequently write "system" when referring to transmitter types, e.g., "glutaminergic system", "octopaminergic system", etc. It I not clear what the term "system" actually refers to. If the authors claim that SIFamide neurons release these transmitters in addition to SIFamide, they should state that precisely and then add experiments to show that this is the case.

**Answer:** We agree with reviewer and removed the word 'system' after the name of neurotransmitter's name.

***Comment 7.*** Figure S6: It is not explained in the figure legend what fly strain "UAS-ctrl" actually is. Does "ctrl" mean control? And what genotype is hat control?
**Answer:** It was wild-type strain. We fixed it as "+".

***Comment 8.*** Figure legend S6, line 1371: The authors indicate experiments using UAS-OrkDeltaC. I could not find these data in the figure.
**Answer:** It's now in Fig.S6U-V.

***Comment 9.*** Line 470: "...reduced branching of SIFa axons at the postsynaptic level" should perhaps be "presynaptic level"?

**Answer:** Reviewer is correct. We fixed it.

***Conclusive Comments:*** *Overall, the study advances our knowledge about the behavioral roles of SIFamide, which is certainly important, interesting, and worthy of being reported. However, the manuscript also raises several serious caveats and includes points that remain speculative and are less convincing. 

Overall, the neuronal basis of action selection based on motivational factors (metabolic state, mating experience, sleep/wake status, etc.) is not well understood. The analysis of SIFamide function in insects might provide a way to address the question how different motivational signals are integrated to orchestrate behavior.*

**Answer:** Thank you for your thoughtful review and for recognizing the significance of our study in advancing knowledge about the behavioral roles of SIFamide. We appreciate your acknowledgment that our work is important, interesting, and worthy of publication.

We understand your concerns regarding the caveats and speculative points raised in the manuscript. We agree that the neuronal basis of action selection influenced by motivational factors—such as metabolic state, mating experience, and sleep/wake status—remains poorly understood. We believe that our analysis of SIFamide function in insects offers valuable insights into how various motivational signals are integrated to orchestrate behavior.

In response to your comments, we have made revisions to clarify our findings and address the concerns raised. We aim to strengthen the arguments presented in the manuscript and provide a more robust discussion of the implications of our results. Thank you once again for your constructive feedback, which has been instrumental in improving the clarity and impact of our work.

**Reviewer #3**

***General Comments:*** *The Manuscript Peptidergic neurons with extensive branching orchestrate the internal states and energy balance of male Drosophila melanogaster by Yuton Song and colleagues addresses the question how SIFamidergic neurons coordinate behavioral responses in a context-dependent manner. In this context the authors investigate how SIFa neurons receive information about the physiological state of the animal and integrate this information into the processing of external stimuli. The authors show that SIFamidergic neurons and sNPPF expressing neurons form a feedback loop in the ventral nerve cord that modulate long mating (LMD) and shorter mating duration (SMD). 

The manuscript is well written and very detailed and provides an enormous amount of data corroborating the claims of the authors. However, before publication the authors may want to address some points of concern that warrant some deeper explanation.*

**Answer:** Thank you for your positive feedback on our manuscript. We appreciate your recognition of the importance of our study in investigating how SIFa neurons integrate information about the physiological state of the animal with external stimuli, as well as your acknowledgment of the substantial data we provide to support our claims. We understand your concerns regarding certain points that require deeper explanation, and we are committed to addressing these issues to enhance the clarity and robustness of our findings. Your insights into the neuronal basis of action selection influenced by motivational factors are invaluable, and we believe that our exploration of SIFamide function in insects contributes significantly to understanding how various motivational signals orchestrate behavior. Thank you once again for your constructive comments, which will help us improve our manuscript before publication.

***Major concerns:***

***Comment 1.*** *On page 6 line 110 the authors describe that knocking-down SIFamide in glia cell does not change LMD or SMD and say that SIFa expression in glia does not contribute to interval timing behavior. However, the authors do not provide any information why they investigate the role of SIFa expression in glia. Is there any SIFa-expression in glia? The authors should somehow demonstrate using antibody labelling against SIFamide whether any glia specific expression of this peptide is to be expected. If they cannot provide this data - the take home message of the experiment cannot be that glia knockdown of SIFamide does not affect the behavior because you cannot knockdown anything that is not there.*

*In the latter case the experiment could be considered as a nice negative control for the elav-Gal4 pan-neuronal knockdown of SIFamide. The authors provide some Figure supplement where they use repo-Gal80 to partially answer this question. However, the authors should keep in mind that Gal4-drivers are not always complete in the expression pattern. Accordingly, the result should be corroborated with immune-labelling against SIFamide directly.*

**Answer:** We appreciate the reviewer's constructive and critical comments regarding the use of our glial cell drivers. As the reviewer rightly pointed out, we believe that glial control is not essential for our manuscript, given that the expression of SIFa is well established in only four neurons. Therefore, we have removed the data related to glial drivers from this manuscript.

***Comment 2.*** *At this point I would like to directly comment on the figure quality. The figures are so crowded that the described anatomical details are hardly visible. In my opinion the manuscript would profit from less data in the main part and more stringent description of the core of the biological problem the authors want to address.* *The authors may want to reduce data from the main text and provide additional data that are not directly related to the main story as supplementary information.*

**Answer:** We agree with the reviewer. As another reviewer also suggested that we streamline our figures and data, we have completely restructured our figures and their presentation. In response, we have significantly reduced the density of the main figures and decreased the size of the graphs to enhance clarity. Additionally, we have increased the spacing between panels to ensure that each component is more easily distinguishable. Further details will be provided in our responses to each comment below.

***Comment 3.*** On page 8 starting with line 140 the authors describe the architecture of SIFamidergic neurons using several anatomical markers e.g., Denmark and further state that they have discovered that the dendrites of SIFa neurons span just the central brain area. Seeing that these data have been published in Martelli et al., 2017 the authors should tune down the claim that this was discovered in their work but rather corroborated earlier results.

**Answer:** We acknowledge this error, as another reviewer also raised this issue. We have corrected our manuscript as follows:

"The established connections and architecture of SIFa neurons has been described by Martelli et al., which enhances our understanding of their functional roles within the neuronal circuitry [51]. To identify the dendritic and axonal components of SIFa-neuronal processes, we employed a similar approach to that reported by Martelli [51]."

***Comment 4.*** *In the next chapter, the authors aim at identifying the presynaptic inputs from SIFa positive neurons that may influence interval timing behavior and make a broad RNAi knock-down screen targeting a majority of neuromodulators. The authors claim that glutaminergic and dopaminergic signaling is necessary for interval timing behavior.* *I guess the authors mean "**glutamatergic" instead of "**glutaminergic" as glutamine is the precursor but not the neurotransmitter.*

**Answer:** The reviewer is correct. We have corrected this error and changed all instances to "glutamatergic."

***Comment 5.*** Furthermore, the authors show that the knock down of Tdc2 with RNAi has comparable effects on SMD than Glutamate and dopamine but appear to not further discuss this in the main text. To me it is not clear why the authors exclude Tdc2 from their resume. The authors should explain this in detail.

**Answer:** We appreciate the reviewer’s constructive comments regarding the need for a more detailed demonstration of the role of Tdc2 data. While we did test Tdc2-RNAi and observed interesting phenotypes, we decided not to include these findings in our publication, as our data on glutamate and dopamine offer a more compelling explanation for how SIFa cotransmission with these neurotransmitters can independently influence various behaviors, such as sleep and mating duration. Consequently, we have removed all data related to Tdc2. We believe that further evaluation is necessary to better understand the roles of the tyramine and octopamine systems in SIFa neurons.

***Comment 6.*** *The authors base their assumptions that the tested neurotransmitters are expressed in SIFamidergic neurons on Scope database analysis. But a transcript does not necessarily mean that it will be translated too. To my knowledge there is no available data in the literature showing that* *tyrosine hydroxylase is expressed in SIFamidergic neurons (see e.g.,* *Mao and Davis, 2010). To show that ple or Tdc2 are indeed expressed and translated into functional enzymes in SIFamidergic neurons the authors should provide the according antibody labelling corroborating the result from the transcriptome analysis.*

**Answer:** We appreciate the reviewer’s constructive comments regarding the role of neurotransmitters in conjunction with SIFa in modulating interval timing behaviors. To confirm the expression of dopamine (DA) in SIFa neurons, we utilized a well-established genetic toolkit for dissecting dopamine circuit function in *Drosophila* [18]. Our findings demonstrate that TH-C-GAL4 specifically labels SIFa neurons, which have been confirmed to be dopaminergic (Fig. S4M). This aligns with the genetic intersection data and the findings from Xie et al. (2018), confirming that a subset of SIFa neurons is indeed dopaminergic. We have included these new results in the main text as follows:

" To further verify the presence of DA neurons within the SIFa neuron population, we utilized a well-established genetic toolkit for dissecting DA circuits and confirmed part of SIFa neurons are dopaminergic (S4M Fig) [58]."

To confirm the glutamatergic characteristics of SIFa neurons, we conducted several experiments that established glutamate as the most critical neurotransmitter for generating interval timing in both SIFa and SIFaR neurons. First, to demonstrate the presence of glutamatergic synaptic vesicles in SIFa neurons, we utilized a conditional glutamatergic synaptic vesicle marker for *Drosophila*, developed by Certel et al. [19]. Our results confirmed that SIFa neurons exhibit strong expression of glutamatergic synaptic vesicles (Fig. 2P and Fig. S4N as a genetic control). We have described these new results in the main text as follows:

"To further the role of glutamate in SIFa-mediated behaviors. we targeted the expression of VGlut receptor in neurons that carry the SIFaR. Strikingly, the knockdown of VGlut receptor in these neurons also disrupted SMD behavior, mirroring the phenotype observed upon direct suppression of glutamatergic signaling in SIFa neurons (S4G-L Fig)."

To further confirm that glutamate release from SIFa neurons influences the function of SIFaR neurons, we tested several RNAi strains targeting glutamate receptors. Our results showed that the knockdown of glutamate receptors in SIFaR-expressing neurons produced phenotypes similar to those observed with VGlut-RNAi knockdown in SIFa neurons (S4G-L Fig). We believe that this series of experiments demonstrates that glutamate and dopamine work in conjunction with SIFa to modulate interval timing and other behaviors related to energy balance. We have described these new results in the main text as follows:

"We also further verified that the knockdown of glutamate receptors in SIFaR-expressing neurons produces phenotypes similar to those resulting from VGlut knockdown in SIFa neurons (S4G-L Fig). This suggests that glutamate is an essential neurotransmitter for modulating interval timing in SIFa neurons."

***Comment 7.*** *The authors compare the LMD and SMD behavior of the animals with reduced expression with "heterozygous control animals" the authors should describe in detail what these are - are these controls the driver lines or the effector lines or a mix of both? The authors should provide the data for heterozygous driver line controls as well as heterozygous effector line controls to exclude any genetic background influence on the measured behavior.* *Accordingly, the authors should provide the data for the same controls for the sleep experiment in figure 3O and all the other behavioral experiments in the following parts of the manuscript.*

**Answer:** We sincerely thank the reviewer for insightful comments regarding the absence of traditional genetic controls in our study of LMD and SMD behaviors. We acknowledge the importance of such controls and wish to clarify our rationale for not including them in the current investigation. The primary reason for not incorporating all genetic control lines is that we have previously assessed the LMD and SMD behaviors of GAL4/+ and UAS/+ strains in our earlier studies. Our past experiences have consistently shown that 100% of the genetic control flies for both GAL4 and UAS exhibit normal LMD and SMD behaviors. Given these findings, we deemed the inclusion of additional genetic controls to be non-essential for the present study, particularly in the context of extensive screening efforts. We understand the value of providing a clear rationale for our methodology choices. To this end, we have added a detailed explanation in the "MATERIALS AND METHODS" section and the figure legends of Figure 1. This clarification aims to assist readers in understanding our decision to omit traditional controls, as outlined below.

"**Mating Duration Assays for Successful Copulation**

The mating duration assay in this study has been reported [33,73,93]. To enhance the efficiency of the mating duration assay, we utilized the Df (1) Exel6234 (DF here after) genetic modified fly line in this study, which harbors a deletion of a specific genomic region that includes the sex peptide receptor (SPR)[94,95]. Previous studies have demonstrated that virgin females of this line exhibit increased receptivity to males [95]. We conducted a comparative analysis between the virgin females of this line and the CS virgin females and found that both groups induced SMD. Consequently, we have elected to employ virgin females from this modified line in all subsequent studies. For naïve males, 40 males from the same strain were placed into a vial with food for 5 days. For single reared males, males of the same strain were collected individually and placed into vials with food for 5 days. For experienced males, 40 males from the same strain were placed into a vial with food for 4 days then 80 DF virgin females were introduced into vials for last 1 day before assay. 40 DF virgin females were collected from bottles and placed into a vial for 5 days. These females provide both sexually experienced partners and mating partners for mating duration assays. At the fifth day after eclosion, males of the appropriate strain and DF virgin females were mildly anaesthetized by CO2. After placing a single female in to the mating chamber, we inserted a transparent film then placed a single male to the other side of the film in each chamber. After allowing for 1 h of recovery in the mating chamber in 25℃ incubators, we removed the transparent film and recorded the mating activities. Only those males that succeeded to mate within 1 h were included for analyses. Initiation and completion of copulation were recorded with an accuracy of 10 sec, and total mating duration was calculated for each couple. All assays were performed from noon to 4pm. Genetic controls with GAL4/+ or UAS/+ lines were omitted from supplementary figures, as prior data confirm their consistent exhibition of normal LMD and SMD behaviors [33,73,93,96,97]. Hence, genetic controls for LMD and SMD behaviors were incorporated exclusively when assessing novel fly strains that had not previously been examined. In essence, internal controls were predominantly employed in the experiments, as LMD and SMD behaviors exhibit enhanced statistical significance when internally controlled. Within the LMD assay, both group and single conditions function reciprocally as internal controls. A significant distinction between the naïve and single conditions implies that the experimental manipulation does not affect LMD. Conversely, the lack of a significant discrepancy suggests that the manipulation does influence LMD. In the context of SMD experiments, the naïve condition (equivalent to the group condition in the LMD assay) and sexually experienced males act as mutual internal controls for one another. A statistically significant divergence between naïve and experienced males indicates that the experimental procedure does not alter SMD. Conversely, the absence of a statistically significant difference suggests that the manipulation does impact SMD. Hence, we incorporated supplementary genetic control experiments solely if they deemed indispensable for testing. All assays were performed from noon to 4 PM. We conducted blinded studies for every test[98,99] .

While we have previously addressed this type of reviewer feedback in our published manuscript [2–7], we appreciate the reviewer’s suggestion to include traditional genetic control experiments. In response, we conducted all feasible combinations of genetic control experiments for LMD/SMD during the revision period. The results are presented in the supplementary figures and are described in the main text.

***Comment 8.*** On page 11 line 231 to page 12 line 233 the authors claim that "sNPF signaling transmits hunger and satiety information to SIFa neurons in order to control food search and feeding" and cite Martelli et al., 2017. Could the authors explain more in detail how the Martelli paper somehow proposes this idea? I do not find the link between sNPF signaling hunger and SIFamide in this precise paper.

**Answer:** We appreciate the reviewer for accurately pointing out our misunderstanding of the references. We agree that Martelli et al.'s paper does not mention that sNPF signaling transmits hunger and satiety information to SIFa neurons. Consequently, we have removed the relevant sentence and replaced it with a statement correctly indicating that while sNPF signaling is related to feeding behavior, its connection to SIFa neurons remains unknown. We are grateful to the reviewer for acknowledging our efforts to accurately cite previous articles that support our rationale and ideas.

" Short neuropeptide F (sNPF) signaling plays a crucial role in regulating feeding behavior in Drosophila melanogaster, influencing food intake and body size [60,66,67] . However, there is currently no direct evidence reported linking sNPF signaling to SIFa neurons."

***Comment 9.*** On page 15 line 302 - 303 the authors write that "except for PK2-R2, all other genes coexpress with SIFa in SCope data, indicating that hugin inputs to SIFa may not be transmitted through peptidergic signaling" - if SIFamidergic neurons do not express hugin-receptors how do the authors explain the inverted effect of PK2-R2-RNAi on single housed male courtship index when compared to heterozygous SIFaPT Gal4 control that show a reduction under comparable conditions.

**Answer:** We appreciate the reviewer’s constructive comments. In line with another reviewer’s suggestion, we have completely removed results of other neuropeptidergic inputs, focusing instead on how sNPF inputs modulate SIFa-mediated behavioral modulation using more advanced techniques such as GCaMP (Fig 3N). Consequently, the phenotypes resulting from various knockdowns of neuropeptide receptors are currently under investigation for a separate manuscript that we are preparing. We hope to successfully address how different neuropeptidergic inputs regulate SIFa neuron activity through various strategies.

***Comment 10.*** *On page 17 line 350 - 351 the authors write that "Stimulation of SIFa neurons resulted in an elevation in food consumption. Further, the authors write that "deactivation of SIFa neurons leads to a decrease in food consumption in male flies".* *From the way this is formulated it is not visible that the role of SIFamide in feeding control was published by Martelli and colleagues before.* *As the authors do not discuss the finding further in their discussion but cite the concerned paper in other aspects it appears as the authors intentionally want to omit this information to the reader. The authors may add a note that this has been shown before for female flies by Martelli and colleagues.*

**Answer:** We appreciate reviewer's concern for properly mention previous Martelli et al.'s results about female feeding behavior modulated by SIFa neurons' activity. We agree with reviewer and added sentence as below in main text.

"Nevertheless, the temporary deactivation of SIFa neurons leads to a decrease in food consumption in male flies (Fig 4N and S6F to S6H) as previously described by Martelli et al.'s report in female flies [43]."

***Comment 11.*** *SIFamide receptor and GnIHR are discussed as descendants from a common ancestor and the authors nicely demonstrate that SIFamide does not only control homeostatic behavior as shown by Martelli and colleagues but also controls reproductive behavior. The evolution of such behavior control mechanisms may be integrated in the discussion too.*	**Answer:** We appreciate the reviewer’s constructive comments, which enhance the evolutionary significance of our study. We agree with the reviewer and have added the following paragraph to the DISCUSSION section:

"The relationship between SIFamide receptors (SIFaR) and gonadotropin inhibitory hormone receptors (GnIHR) [89] highlights an intriguing evolutionary connection, as both are believed to have descended from a common ancestor [90,91]. This study expands on previous findings by Martelli et al., demonstrating that SIFamide not only regulates homeostatic behaviors but also plays a significant role in reproductive behavior [43]. GnIHR regulates food intake and reproductive behavior in opposing directions, thereby prioritizing feeding behavior over other behavioral tasks during times of metabolic need [92]. The evolution of these behavioral control mechanisms suggests a complex interplay between neuropeptides that modulate both physiological states and reproductive strategies. As SIFamide influences various behaviors, including feeding and sexual activity, it may be integral to understanding how organisms adapt their reproductive strategies in response to environmental and internal cues. This integration of behavioral modulation underscores the evolutionary significance of SIFamide signaling in coordinating essential life functions in Drosophila melanogaster and potentially other species, revealing pathways through which neuropeptides can shape behavior across different contexts."

***Conclusive Comments:*** *The manuscript by Song and colleagues is very interesting and may attract a broad readership. However, the authors miss to make clear what was already known and published on the role of SIFamide in homeostatic behavior control before their own study. Seen that the receptors for SIFamide and GnRHI derive from a common ancestor and apparently both GnRHI and SIFamide share similar roles in behavioral control this might indeed suggests that the basic function of this SIFaR/GnIHR-signaling pathway is conserved. This more broad evolutionary aspect is missing in the discussion of the manuscript.*

**Answer:** We wholeheartedly agree with the reviewer regarding the evolutionary significance of SIFaR's function in relation to GnIHR, and we have expanded the DISCUSSION section to emphasize this important aspect.

"The relationship between SIFamide receptors (SIFaR) and gonadotropin inhibitory hormone receptors (GnIHR) [89] highlights an intriguing evolutionary connection, as both are believed to have descended from a common ancestor [90,91]. This study expands on previous findings by Martelli et al., demonstrating that SIFamide not only regulates homeostatic behaviors but also plays a significant role in reproductive behavior [43]. GnIHR regulates food intake and reproductive behavior in opposing directions, thereby prioritizing feeding behavior over other behavioral tasks during times of metabolic need [92]. The evolution of these behavioral control mechanisms suggests a complex interplay between neuropeptides that modulate both physiological states and reproductive strategies. As SIFamide influences various behaviors, including feeding and sexual activity, it may be integral to understanding how organisms adapt their reproductive strategies in response to environmental and internal cues. This integration of behavioral modulation underscores the evolutionary significance of SIFamide signaling in coordinating essential life functions in Drosophila melanogaster and potentially other species, revealing pathways through which neuropeptides can shape behavior across different contexts."

**Reference**

1. Zhang T, Wu Z, Song Y, Li W, Sun Y, Zhang X, et al. Long-range neuropeptide relay as a central-peripheral communication mechanism for the context-dependent modulation of interval timing behaviors. bioRxiv. 2024; 2024.06.03.597273. doi:10.1101/2024.06.03.597273

2. Kim WJ, Jan LY, Jan YN. A PDF/NPF Neuropeptide Signaling Circuitry of Male Drosophila melanogaster Controls Rival-Induced Prolonged Mating. Neuron. 2013;80: 1190–1205. doi:10.1016/j.neuron.2013.09.034

3. Kim WJ, Jan LY, Jan YN. Contribution of visual and circadian neural circuits to memory for prolonged mating induced by rivals. Nat Neurosci. 2012;15: 876–883. doi:10.1038/nn.3104

4. Zhang T, Zhang X, Sun D, Kim WJ. Exploring the Asymmetric Body’s Influence on Interval Timing Behaviors of Drosophila melanogaster. Behav Genet. 2024; 1–10. doi:10.1007/s10519-024-10193-y

5. Sun Y, Zhang X, Wu Z, Li W, Kim WJ. Genetic Screening Reveals Cone Cell-Specific Factors as Common Genetic Targets Modulating Rival-Induced Prolonged Mating in male Drosophila melanogaster. G3: Genes, Genomes, Genet. 2024; jkae255. doi:10.1093/g3journal/jkae255

6. Lee SG, Sun D, Miao H, Wu Z, Kang C, Saad B, et al. Taste and pheromonal inputs govern the regulation of time investment for mating by sexual experience in male Drosophila melanogaster. PLOS Genet. 2023;19: e1010753. doi:10.1371/journal.pgen.1010753

7. Huang Y, Kwan A, Kim WJ. Y chromosome genes interplay with interval timing in regulating mating duration of male Drosophila melanogaster. Gene Rep. 2024; 101999. doi:10.1016/j.genrep.2024.101999

8. Kayser MS, Yue Z, Sehgal A. A Critical Period of Sleep for Development of Courtship Circuitry and Behavior in *Drosophila*. Science. 2014;344: 269–274. doi:10.1126/science.1250553

9. Wong K, Schweizer J, Nguyen K-NH, Atieh S, Kim WJ. Neuropeptide relay between SIFa signaling controls the experience-dependent mating duration of male Drosophila. Biorxiv. 2019; 819045. doi:10.1101/819045

10. Thornquist SC, Langer K, Zhang SX, Rogulja D, Crickmore MA. CaMKII Measures the Passage of Time to Coordinate Behavior and Motivational State. Neuron. 2020;105: 334-345.e9. doi:10.1016/j.neuron.2019.10.018

11. Buhusi CV, Meck WH. What makes us tick? Functional and neural mechanisms of interval timing. Nat Rev Neurosci. 2005;6: 755–765. doi:10.1038/nrn1764

12. Merchant H, Harrington DL, Meck WH. Neural Basis of the Perception and Estimation of Time. Annu Rev Neurosci. 2012;36: 313–336. doi:10.1146/annurev-neuro-062012-170349

13. Allman MJ, Teki S, Griffiths TD, Meck WH. Properties of the Internal Clock: First- and Second-Order Principles of Subjective Time. Annu Rev Psychol. 2013;65: 743–771. doi:10.1146/annurev-psych-010213-115117

14. Rammsayer TH, Troche SJ. Neurobiology of Interval Timing. Adv Exp Med Biol. 2014; 33–47. doi:10.1007/978-1-4939-1782-2_3

15. Golombek DA, Bussi IL, Agostino PV. Minutes, days and years: molecular interactions among different scales of biological timing. Philosophical Transactions Royal Soc B Biological Sci. 2014;369: 20120465. doi:10.1098/rstb.2012.0465

16. Jazayeri M, Shadlen MN. A Neural Mechanism for Sensing and Reproducing a Time Interval. Curr Biol. 2015;25: 2599–2609. doi:10.1016/j.cub.2015.08.038

17. Croset V, Treiber CD, Waddell S. Cellular diversity in the Drosophila midbrain revealed by single-cell transcriptomics. eLife. 2018;7: e34550. doi:10.7554/elife.34550

18. Xie T, Ho MCW, Liu Q, Horiuchi W, Lin C-C, Task D, et al. A Genetic Toolkit for Dissecting Dopamine Circuit Function in Drosophila. Cell Reports. 2018;23: 652–665. doi:10.1016/j.celrep.2018.03.068

19. Certel SJ, Ruchti E, McCabe BD, Stowers RS. A conditional glutamatergic synaptic vesicle marker for Drosophila. G3. 2022;12: jkab453. doi:10.1093/g3journal/jkab453

20. Li H, Janssens J, Waegeneer MD, Kolluru SS, Davie K, Gardeux V, et al. Fly Cell Atlas: A single-nucleus transcriptomic atlas of the adult fruit fly. Science. 2022;375: eabk2432. doi:10.1126/science.abk2432

21. Deng B, Li Q, Liu X, Cao Y, Li B, Qian Y, et al. Chemoconnectomics: Mapping Chemical Transmission in Drosophila. Neuron. 2019;101: 876-893.e4. doi:10.1016/j.neuron.2019.01.045

22. Claridge-Chang A, Assam PN. Estimation statistics should replace significance testing. Nat Methods. 2016;13: 108–109. doi:10.1038/nmeth.3729
