# Supplementary material for: SIFa peptidergic neurons orchestrate the internal states and energy balance of male Drosophila melanogaster
Source: PLoS Biol. 2025 Sep 4;23(9):e3003345. doi: 10.1371/journal.pbio.3003345 (PMC12410877; doi:10.1371/journal.pbio.3003345)
Supplement: Attachment — Submitted filename: Point by point_R2R_PLOS Biology-SIFa-20250707-YS.docx [file pbio.3003345.s019.docx]

**Point-by-point description of the revisions**

**Reviewer #3:**

***Major comments:*** *The manuscript "Peptidergic neurons with extensive branching orchestrate the internal states and energy balance of male Drosophila melanogaster" by Song and colleagues is very interesting for a broad readership. The Authors have substantially addressed the comments raised by the reviewers. However, I still would have some comments before endorsing publication of the manuscript:*
 **Answer:** We sincerely thank the reviewer for their careful re-evaluation of our manuscript and for acknowledging the revisions we made in response to the previous reviewers' comments. We have carefully addressed all the comments raised by the reviewers in our latest revision:

1. **Enhanced Logic & Flow:** We have undertaken a restructuring of the RESULTS and DISCUSSION sections. We have explicitly stated the rationale before each key experiment or set of experiments. The connection between the experimental question, the approach chosen, the results obtained, and the subsequent conclusions drawn is now clear and unambiguous throughout the manuscript. Transitions between sections have also been strengthened to improve the overall logical progression.
2. **Strengthened Support for Conclusions:** We have meticulously reviewed all stated conclusions, especially those highlighted as potentially unsupported. For each conclusion, we have explicitly referenced the specific data figures/panels that support it, ensured the data presented is robust and directly relevant to the claim, toned down any language that could over-interpret the data, and more clearly acknowledged limitations or alternative interpretations where appropriate.

We believe these substantial revisions have significantly enhanced the manuscript's clarity, logical flow, and the robustness with which conclusions are supported by the data. We hope this revised version fully addresses the reviewer's valid concerns.

Below we address each comment point-by-point, with all corresponding revisions highlighted in the updated manuscript.

***Comment 1.*** *During the review process the authors were asked to show evidence that SIFamdergic neurons indeed express tyrosine hydroxylase. The authors show the expression of ple through Scope RNA seq data (Figure 1O, related to page 10, line 208) and indirectly by employing an expression toolkit for dopaminergic neurons. There are very reliable and well described antibodies against Tyrosine hydroxylase commercially available.* *In my opinion direct proof that SIFamide neurons are dopaminergic through anti-TH immunoreactivity (immunostar) would be important.*
 **Answer:** Thank you for your insightful comment regarding the direct proof of SIFamide neurons being dopaminergic. To address this point, we have conducted several experiments.

1. We have utilized the **Dop1R1-Tango** system [1], which generates a GFP signal upon dopamine binding, and observed GFP expression in SIFaR cells, indicating dopamine signaling in these neurons (Fig 3A and S5I). We have incorporated our new findings obtained using Dop1R1-Tango into the main text, as detailed below.

“To verify the presence of dopaminergic (DA) neurons within the SIFa neuron population, we utilized the Dop1R1-Tango system, which produces a GFP signal upon dopamine binding [59]. Our results confirm the existence of active dopamine receptors in SIFaR neurons and demonstrate that these neurons are responsive to dopamine (Fig 3A; genetic control shown in S5I Fig). Given that SIFaR^24F06^ neurons have been identified as forming direct synaptic connections with SIFa neurons (Fig 3A), these findings substantiate that SIFa neurons employ DA signaling to modulate SIFaR-expressing neural circuits (Zhang 2024).” (L257-264)

2. Additionally, we performed RNAi experiments targeting various dopamine receptors in SIFaR cells and found that Dop1R1-RNAi specifically affected SMD behavior, while other receptors' RNAi influenced both SMD and LMD behaviors (Fig. S5 A-H). These results provide additional evidence for the dopaminergic nature of SIFa neurons and their role in modulating specific behaviors. We have described these results in main text as below:

“Additionally, we conducted RNAi experiments targeting Dop1R1, Dop1R2, DopEcR, and Dop2R receptors in SIFaR-expressing cells to elucidate the role of DA signaling in modulating interval timing mediated by SIFa neurons (S5A–H Fig). Our findings indicated that *Dop1R1-RNAi* selectively influenced SMD behavior. Conversely, the other dopamine receptors, when diminished, affected both SMD and LMD behaviors. These results indicate that diverse dopamine receptors have specific functions in modulating interval timing behaviors.” (L265-271)

3. Furthermore, we conducted immunofluorescence colocalization experiments using *TH-C-GAL4*, *TH-F-GAL4* with *GAL4^SIFa.PT^*, and found partial colocalization between *TH-C-GAL4*/*TH-F-GAL4* and SIFa neurons (S5J-K Fig). This is consistent with the study by Xie et al.[2] , where they used *TH-C-GAL4*, *TH-D-GAL4*, and *TH-F-GAL4* drivers and stained with TH antibodies. In their work, *TH-C-GAL4* labeled cells that were suspected to be SIFa neurons and stained by TH antibody, while TH-F-GAL4 also labeled cells suspected to be SIFa neurons but were not stained by the TH antibody. The partial colocalization we observed between *TH-C-GAL4*/*TH-F-GAL4* and *GAL4^SIFa.PT^* further supports the dopaminergic identity of a subset of SIFa neurons. We have described these results in main text as below:

“We also employed a well-established genetic toolkit to analyze dopaminergic (DA) circuits and confirmed that a subset of SIFa neurons is dopaminergic (S5J-K Fig) [61].” (L271-273)

4. We also examined the recently published fly brain connectome dataset in Flywire and confirmed that SIFa neurons include dopaminergic neurons, which we have detailed in the main text as follows:

“This finding aligns with predictions from Flywire data, which indicate that SIFa neurons include dopaminergic neurons [62,63].”

5. We utilized the next-generation GRAB sensor developed for *in vivo* monitoring of dopaminergic activity (Sun 2020). Specifically, we selectively activated SIFa neurons using the *UAS-P2X2* system by applying ATP (Jiang 2011,Hu 2010), and subsequently monitored the GRAB sensor signals in SIFaR neurons. Consistent with our previous findings indicating that SIFa neurons contain a dopaminergic system, the GRAB sensor in SIFaR neurons showed responses upon ATP-induced activation of P2X2-expressing SIFa neurons (Fig 3B). Taken together, these results strongly support our conclusion that SIFa neurons employ the dopaminergic system to convey internal states, thereby modulating context-dependent interval timing behaviors.

The convergence of our GRAB sensor data, P2X2 activation studies, and connectome analysis provides multiple independent lines of evidence supporting the dopaminergic capacity of SIFa neurons. This multi-faceted approach addresses concerns about the specificity and physiological relevance of dopaminergic signaling in SIFa neurons, demonstrating that these neurons employ dopaminergic co-transmission alongside their established neuropeptidergic functions to orchestrate complex behavioral modulation, particularly in the domain of context-dependent interval timing. We have described these results in main text as below:

“To further validate our hypothesis that SIFa neurons utilize the DA system for neurotransmission, we employed next-generation GRAB (GPCR-activation-based) sensors specifically developed for monitoring dopaminergic activity *in vivo* (Sun 2020,Zhuo 2024). These sensors represent a significant advancement in neurotransmitter detection, offering high sensitivity, selectivity, and signal-to-noise properties with subsecond response kinetics and the ability to detect a wide range of dopamine concentrations (Sun 2020,Zhuo 2024). We implemented a targeted experimental approach using the *UAS-P2X2* system to achieve precise activation of SIFa neurons through ATP application (Jiang 2011,Hu 2010). This system enables specific neuronal activation while maintaining temporal precision, as P2X2 receptors are ATP-gated ion channels that generate rapid, non-specific cation conductance upon ATP binding. Our experimental design involved expressing GRAB sensors in SIFaR neurons while simultaneously expressing P2X2 receptors in SIFa neurons. Upon ATP application to activate the P2X2-expressing SIFa neurons, we monitored the resulting DA signals through the GRAB sensor responses in downstream SIFaR neurons. As predicted by our previous experimental evidence supporting the presence of dopaminergic components within SIFa neurons, the GRAB sensors exhibited activation in SIFaR neurons following ATP-induced stimulation of SIFa neurons (Fig 3B-D).

This response pattern provides compelling evidence for several key aspects of SIFa neurotransmission: First, it confirms functional dopamine release from SIFa neurons upon activation. Second, it demonstrates the existence of functional dopaminergic signaling pathways from SIFa to SIFaR neurons. Third, it validates the physiological relevance of dopaminergic co-transmission in SIFa neurons, complementing their known neuropeptidergic signaling. Our demonstration that SIFa neurons can release dopamine positions them as critical modulators in timing circuits, capable of transmitting internal state information that influences context-dependent interval timing behaviors.” (L276-303)

***Comment 2.*** *Further, in Figure 1L the authors the expression pattern of SIFa.PT-Gal4. The images on the left - both, the overview and the magnification the image shows some bizarre lines on the upper left part of the brain - the authors should improve the image quality.*

**Answer:** Thank you very much for your insightful comments on our manuscript, especially regarding the image quality of Figure 1L (now Fig.1Q). We deeply appreciate your time and effort in reviewing our work.

We have carefully examined the noise issue you pointed out in the upper left part of the brain image. After thorough discussion and re-evaluation, we believe that this noise may be due to some technical limitations during the image acquisition process. However, we want to emphasize that this noise does not affect the overall interpretation and conclusion of the expression pattern of *GAL4^SIFa.PT^*.

To address your concern and enhance the clarity of the figure, we have marked the noise area in the image. This is to help readers better distinguish the specific signal from the background noise. We have also adjusted the overall signal intensity to reduce the prominence of the noise as much as possible while preserving the essential information of the expression pattern.

***Comment 3.*** *In line 132 on page 6 - the authors write that SIFa "protein" level remained the same - seen the size of SIFamide it would be preferable if the authors stick to the term peptide instead of protein.*

**Answer:** Thank you for bringing this to our attention. We appreciate your suggestion to use the term "peptide" instead of "protein" when referring to SIFamide, particularly given its size. We have revised the text in line 132 on page 6 accordingly, replacing "protein" with "peptide" to more accurately describe SIFamide.

***Comment 4.*** *Lastly, it would be preferable that on page 18, line 360 the authors again refer to Martelli et al., as this reflects exactly the same experiment that was conducted by this group.*

**Answer:** We agree with the reviewer and have included Martelli et al. in the main text where we discuss feeding behavior.

**Reviewer #4:**

***Major comments:*** *This manuscript by Song et al focuses on SIFa and its role in context-dependent mating duration. The authors show that feedback between sNPF and SIFa is important for this behaviour. Overall, I found it challenging to follow all the experiments presented in this manuscript. It was not always clear the motivation for performing some experiments. In other instances, unusually large amount of attention is given to experiments that seem superfluous e.g. knockdown of SIFa in glia or re-characterizing the morphology of SIFa neurons which don't add much to the overall message of the manuscript. While the authors have addressed some of the concerns raised by previous reviewers, I think that the overall message of the manuscript is hard to follow This is not made easy by the fact that the genetic controls are not presented with the experimental treatments in the same figures.* *The manuscript still needs extensive restructuring so that main message of the manuscript becomes clear.*	**Answer:** Thank you for your thoughtful feedback and the time invested in evaluating our manuscript. We sincerely appreciate your concerns regarding clarity, motivation for specific experiments, and the presentation of genetic controls. We have carefully addressed these points in the revised manuscript, guided by your suggestions and the editorial team’s recommendations. Below, we detail the specific changes implemented to enhance clarity and focus:

**1. Integration of Genetic Controls into Main Figures:** We agree that co-localizing genetic controls with experimental data in the same figures improves readability. To address this:

- Figures S6B–D (controls for SIFa rescue experiments) have been moved to Figure 5H–I and 5N.

- Figures S5N–S (controls for neuronal manipulations) are now integrated into Figure 4M–R.

- Figures S8G–J remain in the supplement (as they duplicate data in Figure S7), but we added cross-references to streamline navigation.

These adjustments ensure that controls are visually aligned with their corresponding results, reducing cognitive load for readers.

**2. Streamlining of Peripheral Experiments**: We acknowledge your concern about the depth of certain experiments (e.g., SIFa knockdown in glia, neuronal morphology re-analysis). While these data provide mechanistic context (e.g., confirming cell-type specificity and ruling off-target effects), we have:

- Shortened text descriptions of these experiments.

- Moved genetic control data (Figure S2A–H) to Figure 1I-P, consolidating genetic controls with experimental treatments as reviewer suggested.

- Relocated Figure 1J and 1M to Figure S1D and E, as they are supplemental rather than central to the main narrative.

This reorganization prioritizes the core message—sNPF/SIFa feedback in mating duration—while retaining supplemental data for rigor.

**3. Enhanced Narrative Clarity:** To improve logical flow:

- Motivations for experiments are explicitly stated in the Results section (e.g., "To test whether X mediates Y, we performed Z").

- Superfluous details (e.g., glial knockdown rationale) were condensed or moved to the supplement.

- The DISCUSSION now explicitly links each experiment to the central hypothesis, avoiding tangential threads.

**4. Alignment with Editorial Guidance**: While the Academic Editor and Editor-in-Chief deemed the original structure acceptable, your feedback highlighted opportunities for targeted improvements. We focused revisions on:

- Figure consolidation (to unify controls/results).

- Text streamlining (to emphasize the feedback mechanism’s role in mating behavior).

- Improved signposting (e.g., subsection headers, figure captions) to guide readers through the logic.

Your insights significantly strengthened the manuscript, and we hope the revised version alleviates the concerns raised. All changes were made in consultation with the editorial team to balance clarity with scientific completeness. We remain open to further adjustments if specific sections still require refinement. Thank you again for your invaluable input.

***Major Comment 1.*** *Not all neurons in TH-C GAL4 are anti-TH positive. The authors can refer to the original paper by Xie et al where this line was characterized. As suggested by previous reviewers, stronger evidence is needed for the presence of dopamine in SIFa neurons.*
 **Answer:** Thank you very much for your insightful and constructive comments on our study regarding the dopaminergic nature of SIFamide neurons. We sincerely appreciate your suggestions and the time you have taken to evaluate our work. In response to your queries and to further strengthen the evidence for the dopaminergic identity of SIFa neurons, we have conducted a series of additional experiments. Since **Reviewer #3** raised the same concern about the involvement of the DA system in SIFa signaling, we have provided the same response here to address our additional experiments supporting the role of SIFa neurons in using the DA system to modulate interval timing behaviors.

1. We have utilized the **Dop1R1-Tango** system [1], which generates a GFP signal upon dopamine binding, and observed GFP expression in SIFaR cells, indicating dopamine signaling in these neurons (Fig 3A and S5I). We have incorporated our new findings obtained using Dop1R1-Tango into the main text, as detailed below.

“To verify the presence of dopaminergic (DA) neurons within the SIFa neuron population, we utilized the Dop1R1-Tango system, which produces a GFP signal upon dopamine binding [59]. Our results confirm the existence of active dopamine receptors in SIFaR neurons and demonstrate that these neurons are responsive to dopamine (Fig 3A; genetic control shown in Fig. S5I). Given that SIFaR^24F06^ neurons have been identified as forming direct synaptic connections with SIFa neurons (Fig 3A), these findings substantiate that SIFa neurons employ DA signaling to modulate SIFaR-expressing neural circuits (Zhang 2024).” (L257-264)

2. Additionally, we performed RNAi experiments targeting various dopamine receptors in SIFaR cells and found that Dop1R1-RNAi specifically affected SMD behavior, while other receptors' RNAi influenced both SMD and LMD behaviors (Fig. S5 A-H). These results provide additional evidence for the dopaminergic nature of SIFa neurons and their role in modulating specific behaviors. We have described these results in main text as below:

“Additionally, we conducted RNAi experiments targeting Dop1R1, Dop1R2, DopEcR, and Dop2R receptors in SIFaR-expressing cells to elucidate the role of DA signaling in modulating interval timing mediated by SIFa neurons (Fig. S5A–H). Our findings indicated that *Dop1R1-RNAi* selectively influenced SMD behavior. Conversely, the other dopamine receptors, when diminished, affected both SMD and LMD behaviors. These results indicate that diverse dopamine receptors have specific functions in modulating interval timing behaviors.” (L265-271)

3. Furthermore, we conducted immunofluorescence colocalization experiments using *TH-C-GAL4*, *TH-F-GAL4* with *GAL4^SIFa.PT^*, and found partial colocalization between *TH-C-GAL4*/*TH-F-GAL4* and SIFa neurons (S5J-K Fig). This is consistent with the study by Xie et al.[2] , where they used *TH-C-GAL4*, *TH-D-GAL4*, and *TH-F-GAL4* drivers and stained with TH antibodies. In their work, *TH-C-GAL4* labeled cells that were suspected to be SIFa neurons and stained by TH antibody, while TH-F-GAL4 also labeled cells suspected to be SIFa neurons but were not stained by the TH antibody. The partial colocalization we observed between *TH-C-GAL4*/*TH-F-GAL4* and *GAL4^SIFa.PT^* further supports the dopaminergic identity of a subset of SIFa neurons. We have described these results in main text as below:

“We also employed a well-established genetic toolkit to analyze dopaminergic (DA) circuits and confirmed that a subset of SIFa neurons is dopaminergic (Fig. S5J-K) [61].” (L271-273)

4. We also examined the recently published fly brain connectome dataset in Flywire and confirmed that SIFa neurons include dopaminergic neurons, which we have detailed in the main text as follows:

“This finding aligns with predictions from Flywire data, which indicate that SIFa neurons include dopaminergic neurons [62,63].”

5. We utilized the next-generation GRAB sensor developed for *in vivo* monitoring of dopaminergic activity (Sun 2020). Specifically, we selectively activated SIFa neurons using the *UAS-P2X2* system by applying ATP (Jiang 2011,Hu 2010), and subsequently monitored the GRAB sensor signals in SIFaR neurons. Consistent with our previous findings indicating that SIFa neurons contain a dopaminergic system, the GRAB sensor in SIFaR neurons showed responses upon ATP-induced activation of P2X2-expressing SIFa neurons (Fig 3B). Taken together, these results strongly support our conclusion that SIFa neurons employ the dopaminergic system to convey internal states, thereby modulating context-dependent interval timing behaviors.

The convergence of our GRAB sensor data, P2X2 activation studies, and connectome analysis provides multiple independent lines of evidence supporting the dopaminergic capacity of SIFa neurons. This multi-faceted approach addresses concerns about the specificity and physiological relevance of dopaminergic signaling in SIFa neurons, demonstrating that these neurons employ dopaminergic co-transmission alongside their established neuropeptidergic functions to orchestrate complex behavioral modulation, particularly in the domain of context-dependent interval timing. We have described these results in main text as below:

“To further validate our hypothesis that SIFa neurons utilize the DA system for neurotransmission, we employed next-generation GRAB (GPCR-activation-based) sensors specifically developed for monitoring dopaminergic activity *in vivo* (Sun 2020,Zhuo 2024). These sensors represent a significant advancement in neurotransmitter detection, offering high sensitivity, selectivity, and signal-to-noise properties with subsecond response kinetics and the ability to detect a wide range of dopamine concentrations (Sun 2020,Zhuo 2024). We implemented a targeted experimental approach using the *UAS-P2X2* system to achieve precise activation of SIFa neurons through ATP application (Jiang 2011,Hu 2010). This system enables specific neuronal activation while maintaining temporal precision, as P2X2 receptors are ATP-gated ion channels that generate rapid, non-specific cation conductance upon ATP binding. Our experimental design involved expressing GRAB sensors in SIFaR neurons while simultaneously expressing P2X2 receptors in SIFa neurons. Upon ATP application to activate the P2X2-expressing SIFa neurons, we monitored the resulting DA signals through the GRAB sensor responses in downstream SIFaR neurons. As predicted by our previous experimental evidence supporting the presence of dopaminergic components within SIFa neurons, the GRAB sensors exhibited activation in SIFaR neurons following ATP-induced stimulation of SIFa neurons (Fig 3B-D).

This response pattern provides compelling evidence for several key aspects of SIFa neurotransmission: First, it confirms functional dopamine release from SIFa neurons upon activation. Second, it demonstrates the existence of functional dopaminergic signaling pathways from SIFa to SIFaR neurons. Third, it validates the physiological relevance of dopaminergic co-transmission in SIFa neurons, complementing their known neuropeptidergic signaling. Our demonstration that SIFa neurons can release dopamine positions them as critical modulators in timing circuits, capable of transmitting internal state information that influences context-dependent interval timing behaviors.” (L276-303)

***Major Comment 2.*** *How can the authors explain the fact that synaptic transmission inhibition of SIFa neurons using Shibire has no effect on mating duration but Vglut, ple or SIFa knockdown in SIFa neurons all have an effect. Surely, inhibition of the neurons is a more drastic manipulation than knockdown of each neurotransmitter.*
 **Answer:** Thank you for this insightful question, which highlights a nuanced aspect of our study. We agree that the differential effects of shibire^ts^ (temperature-sensitive Dynamin mutant of *Drosophila*, synaptic transmission blockade) versus neurotransmitter/neuropeptide knockdowns (Vglut-, ple-, SIFa-RNAi) warrant clarification. We address this point below, integrating established neurobiological principles with our data:

**1. Differential Targeting of Vesicular Release Mechanisms**: SIFa neurons likely utilize dual neurotransmission:

- Fast synaptic transmission (glutamate via Vglut-dependent small clear vesicles, SCVs).

- Neuropeptide release (SIFa via dense-core vesicles, DCVs): While shibire^ts^ blocks SCV recycling and acute synaptic transmission (e.g., glutamate) (Kroll 2015), it does not seriously disrupt DCV-mediated neuropeptide release although there is attention that synaptic neuropeptide release by dynamin-dependent partial release from circulating vesicles (Wong 2015). Crucially:

- SIFa signaling highly depends on DCVs, but requires SCVs (supported by our experiments).

- *Vglut-RNAi* depletes glutamate in SCVs, eliminating fast transmission of glutamatergic signaling completely.

- *ple-RNAi* depletes dopamine in SCVs, eliminating fast transmission of dopaminergic signaling completely.

- *SIFa-RNAi* depletes the neuropeptide itself in DCVs.

Thus, shibire^ts^ only inhibits the fast component partially, while RNAi seriously disrupts the neuropeptide and neurotransmitter components essential for interval timing modulation. This hypothesis could be further strengthened by the subsequent hypothesis comparing chronic and acute effects.

**2. Chronic vs. Acute Manipulations**

- shibire^ts^: Temperature-dependent, acute, and reversible synaptic blockade. Neuropeptide stores (DCVs) remain intact and may still be released via activity-independent mechanisms.

- RNAi: Chronic depletion (days-weeks) of neurotransmitters/neuropeptides, causing irreversible loss of function.

This explains why only chronic disruption of SIFa/Vglut/ple—not acute synaptic inhibition—impairs mating behavior.

**3. Compensatory Mechanisms in shibire^ts^:** Neuropeptide release can occur independently of shibire^ts^-sensitive SV recycling. SIFa neurons might compensate for lost fast transmission via enhanced neuropeptide secretion or parallel signaling pathways during shibire^ts^ experiments (Jing 2010).

**4. Dual-Transmitter Architecture Enables Behavioral Specificity**: The differential effects of shibire^ts^ on feeding versus mating behaviors provide critical insight into the functional versatility of SIFa neurons (Fig 5L-N vs. S7E-H Fig). We propose that SIFa neurons employ behavior-specific transmission mechanisms:

- Feeding (S7E-H Fig) requires fast synaptic transmission (likely glutamate-mediated via small clear vesicles, SCVs), which is disrupted by shibire^ts^ due to its blockade of dynamin-dependent SCV recycling.

- Mating duration, however, depends on neuropeptide release (SIFa via dense-core vesicles, DCVs), which is shibire^ts^-resistant but impaired by chronic depletion of SIFa, ple, or Vglut.

This dichotomy explains why shibire^ts^ affects feeding but not mating: Feeding relies on rapid SCV-mediated signaling, while mating involves sustained DCV-mediated neuromodulation insensitive to acute synaptic blockade. Chronic loss of Vglut (disrupting SCV glutamate), ple (blocking neuropeptide processing), or SIFa (depleting DCV cargo) all compromise the neuropeptide-dependent mating pathway.

Thus, SIFa neurons utilize parallel signaling modes—fast neurotransmission for rapid behaviors (feeding) and activity-independent neuropeptide release for prolonged modulation (mating). This functional segregation allows one neuronal population to regulate diverse behaviors without crosstalk. We have expanded this DISCUSSION to clarify this concept.

We sincerely appreciate your question, which allowed us to clarify this critical mechanistic distinction and strengthen the narrative. Your expertise has been invaluable in highlighting nuances that enrich the study’s impact. Should further elaboration be needed, we welcome the opportunity to discuss this in greater detail.

“The absence of mating defects following acute synaptic inhibition of SIFa neurons (shi^ts^) contrasted with the impairments observed upon chronic knockdown of *Vglut*, *ple*, or SIFa itself, reveals a fundamental characteristic of SIFa neuron function (Fig 5L-N vs. S7E-H Fig). We propose that these neurons employ dual transmission modes: fast synaptic glutamate signaling (mediated by small clear vesicles, SCVs) and activity-independent neuropeptide release (via dense-core vesicles, DCVs). While shibir^ts^ disrupts SCV recycling and acute synaptic transmission (Kroll 2015), it spares DCV-mediated SIFa neuropeptide secretion. Conversely, chronic depletion of glutamate (*Vglut-RNAi*), neuropeptide processing (*ple-RNAi*), or SIFa itself eliminates essential components for sustained neuromodulation. This distinction highlights that mating duration regulation depends predominantly on neuropeptide-mediated signaling rather than fast neurotransmission, aligning with established DCV resilience to dynamin-dependent endocytosis blockade (Wong 2015). Thus, the phenotype manifests only when the neuropeptide pathway is chronically compromised. This mechanistic segregation explains why acute synaptic blockade (shi^ts^) disrupts feeding but not mating — shi^ts^ impair SCV recycling but spares DCV exocytosis. Conversely, chronic depletion of SIFa, ple, or Vglut compromises mating by directly or indirectly disabling neuropeptide signaling. These findings highlight a functional division of labor within SIFa neurons, where SCVs drive rapid behaviors (e.g., feeding) and DCVs mediate sustained neuromodulation (e.g., mating). Such multiplexed signaling enables a single neuronal population to coordinate diverse physiological outputs without behavioral interference, underscoring the adaptability of peptidergic circuits in encoding context-dependent responses.” (L535-557)

***Major Comment 3.*** *FlyScope data are not convincing. Multiple markers are necessary to conclusively claim that the transcriptomes of interest belong to a particular cell type. Neuropeptide transcripts tend to be expressed in very high amounts and it can generate false positives (see:* *https://pubmed.ncbi.nlm.nih.gov/32314735/ ).*

**Answer:** Thank you for your feedback on our manuscript, particularly regarding the FlyScope data. We understand your concerns about the convincingness of the FlyScope data and the need for multiple markers to conclusively identify cell types. We completely agree with the reviewer on this point.

In response to your comments, we have carefully re-examined our analysis and presentation of the single RNA sequencing data from FlyScope. In our revised manuscript, we have taken a more cautious approach to describing the FlyScope data as below in main text:

“Fly Scope single-cell RNA sequencing data suggest potential high-level co-expression of *VGlut* and *ple* in SIFa neurons (Fig 2N-O).” (L256-257)

We have retained the tSNE plots (Fig 2N-O) to illustrate the potential co-expression of *ple* and *Vglut* genes in SIFa cells, but we have modified the text to clarify that this is an analysis that complements our main experimental results.

***Major Comment 4.*** *Figure 5A-E - the confocal images look oversaturated. I am not sure how one can accurately quantify the area of fluorescence based on these.*

**Answer:** Thank you for your feedback on our manuscript and for pointing out the issue of oversaturation in the confocal images of Figure 5A-E. We sincerely appreciate your concerns regarding the quantification of fluorescence areas.

We have taken your comments seriously and have reprocessed the images to reduce the overexposure to the best of our ability while still clearly showing the relevant features. We want to emphasize that even with the initial oversaturation, the differences in fluorescence intensity between the experimental groups were clearly distinguishable and statistically significant. Our quantification methodology focuses on the relative differences in fluorescence signals across the groups rather than relying solely on the absolute intensity values. This approach has allowed us to reliably assess the differences in fluorescence areas and supports the conclusions drawn in our study.

***Specific comments***
***Comment 1.*** *Figure 1A: is it not significant or is there a difference*

**Answer:** Thank you for your careful review of our manuscript and for pointing out the issue in Figure 1A. We sincerely apologize for the confusion caused by the incorrect labeling of "ns".

We have now corrected the figure, ensuring that the significance level is accurately represented with the appropriate four-star marker. The revised Figure 1A correctly displays the statistically significant difference, which aligns with our analysis and the results described in the text.

***Comment 2.*** *Lines 114-116: I am not sure why this experiment is important since several previous studies have conclusively showed that SIFa is expressed in neurons and not glia.*
 **Answer:** Thank you for your feedback on our manuscript. We acknowledge your point that previous studies have already established that SIFa is expressed in neurons and not glia.

In response to your comment, we have revised the manuscript by moving the relevant data from the main figures to the supplementary figure (Figure S1A). This change ensures that the focus of the manuscript remains on our novel findings while still providing the supporting data for the paper.

We appreciate your guidance in refining our manuscript to better emphasize our new contributions to the field.

***Comment 3.*** *Lines 121-124: Since the authors don't show staining pattern for the GAL4 for the rest of the brain and VNC, it is difficult to conclude that the phenotype is due to SIFa knockdown in only the intended neuronal population. Figure S1B shows SIFa staining in several neurons in w1118 flies. It is certainly expressed in more than 4 neurons.*

**Answer:** Thank you for raising this important point regarding the need for comprehensive GAL4 characterization. We fully agree that validating the spatial restriction of genetic manipulations is essential for precise phenotypic attribution. To address your concern directly:

1. We have now included a new whole-brain image in Figure S1C (formerly S1B, revised per your suggestion), demonstrating the expression pattern of the SIFa peptide by SIFa-RNAi with different drivers used throughout our study.
2. As shown in Figure S1C, endogenous SIFa is broadly expressed in the *w^1118^* control. However, SIFa antibody signals are specifically reduced by SIFa-RNAi knockdown driven by *GAL4^SIFa.PT^* and *elav^c1555^*, but not by *repo-GAL4* mediated knockdown. This indicates that our antibody staining specifically labels neuronal SIFa peptide.
3. As the reviewer pointed out, the *w^1118^* staining appears to highlight several neurons. This is because the SIFa peptide is distributed along the entire length of SIFa axons and dendritic branches throughout the brain. A similar distribution pattern has been previously reported by another group (Terhzaz 2007). (See below image included in this document)

We regret this critical validation image was not initially included and appreciate your guidance in strengthening this aspect of the manuscript. The updated figure definitively resolves the concern about phenotypic specificity.

***Comment 4.*** *Figure S1C: SIFa staining looks unlike what has been reported previously. The authors also state that these neurons have extensive branching but I don't see any branching in their images. Given the quality of this imaging, it is difficult to make any conclusions on the SIFa protein levels.*

**Answer:** We thank the reviewer for their careful assessment of Figure S1C (which is now Figure S1D) and the valid points raised regarding the SIFa staining pattern and image quality. We address these concerns below:

**1. Difference in Staining Pattern:** The observed staining pattern in Figure S1D differs from previously reported patterns because we utilized a novel, custom-generated SIFa polyclonal antiserum in this study. It is well-established that different polyclonal antibodies raised against the same antigen can exhibit distinct binding affinities and recognize different epitopes, leading to variations in staining intensity, cellular localization (e.g., cell body vs. process dominance), and overall pattern. Our antiserum represents a unique immunological reagent compared to those used in prior studies.

**2. Specificity and Validation of Our Antiserum:** While we acknowledge that our antiserum produces a weaker overall signal compared to some previously reported antibodies, we have rigorously confirmed its specificity for SIFa neurons:

- Cell Body Labeling: The antiserum clearly and specifically labels SIFa neuronal cell bodies.

- Neuronal Specificity: Staining is restricted to neuronal structures.

- Functional Validation: Crucially, staining is significantly reduced or abolished in well-characterized *SIFa-GAL4* neurons following targeted SIFa knockdown (using *SIFa-RNAi*), demonstrating the antibody's dependence on the presence of the SIFa protein. This knockdown control strongly supports the antigen-specific reactivity of our antiserum.

**3. Branching Morphology:** The reviewer notes difficulty in observing the extensive branching we describe. We agree that the lower signal intensity of our novel antiserum, particularly within the fine neuronal processes, makes the branching morphology less prominent in standard micrographs compared to images generated with stronger antisera. While the cell bodies are unequivocally labeled, the processes are indeed fainter. We focused our analysis on the robust cell body labeling for quantification and identification. We acknowledge that the current image quality limits the visualization of finer processes clearly.

**4. Conclusion on SIFa Levels:** While the weaker signal intensity of our novel antibody presents imaging challenges, the specificity validation (especially the knockdown experiment) gives us confidence that the cell body labeling accurately reflects SIFa expression. The different staining pattern observed (fainter processes, clearer cell bodies) is therefore primarily attributable to the distinct characteristics of our custom-generated polyclonal antiserum compared to reagents used by other groups, rather than non-specific staining or poor quality per se. We believe the data obtained with this validated antibody remain interpretable for the specific conclusions drawn regarding SIFa neuronal cell body identification and their response to our experimental manipulations. We have added the following sentence to clarify for readers that our staining patterns differ from those reported in previous studies.

“Notably, our custom-generated SIFa polyclonal antiserum, while exhibiting weaker overall signal intensity compared to previously reported antibodies (Terhzaz 2007), specifically labels SIFa neuronal cell bodies and is validated by SIFa-GAL4-mediated knockdown.” (line127-130)

***Comment 5.*** *Lines 143-145:* *The representative images don't match the conclusion. It looks like the staining in males is more intense and there is increased arborization in female antennal lobes. So I am not entirely convinced by the conclusions of this analysis.*

**Answer:** We appreciate the reviewer's careful observation regarding the representative images in Figure 1J-K and quantification data in Figure S1G-H. Although we agree with the reviewer's qualitative assessment that staining appears more intense in males and suggests increased arborization in female antennal lobes, our quantitative analysis using ImageJ (measuring immunoreactive area coverage in whole brain regions showed no statistically significant differences. Given this quantitative result and to avoid overinterpretation, we have limited our findings in the main text to the following descriptive observation:

“While qualitative observations suggest more intense SIFa immunoreactivity in males and increased arborization patterns in female antennal lobes, quantitative analysis showed no significant differences in regional coverage (S1I-J Fig). Precise morphological investigation would be necessary to discern potential sexual dimorphic differences in SIFa neuronal architecture.” (line152-157)

***Comment 6.*** *Lines 147-153: The motivation for performing these experiments is not provided so the reader is left confused.*
**Answer:** Thank you for your comments on our manuscript. We understand your concern regarding the motivation for the experiments described in lines 147-153.

In this section, our goal was to explore whether SIFa neurons play a role in sexual dimorphism. We were particularly interested in determining if SIFa neurons contribute to male-specific behaviors. To address this, we expressed feminizing genes such as *UAS-dsx^F^*, *UAS-tra^F^*, and *UAS-sxl* in SIFa neurons using the *GAL4^SIFa.PT^* driver. By doing so, we aimed to assess whether altering the sexual characteristics of SIFa neurons would influence interval timing behaviors.

We appreciate that the original text may not have clearly conveyed this motivation. To clarify, we have revised the text as below:

“Building on this foundation, we sought to determine if the sexual dimorphism in SIFa neurons contributes to the regulation of interval timing behaviors. We have validated that *GAL4^SIFa.PT^* neurons do not label *fru^FLP^*-positive neurons in the male brain (S1J Fig). Interval timing behaviors are unaffected by the expression of female form of *doublesex,* which drives female-specific splicing of downstream genes (*UAS-dsx^F^*) (S1K and S1L Fig); *transformer*, a key player in female sex determination affecting dsx splicing (*UAS-tra^F^*) (S1M and S1N Fig); or *sex lethal* (*UAS-sxl*) (S1O and S1P Fig) all of which have strong feminizing activity. According to these results and genetic control experiments (S2A-H Fig), neurons that express SIFa are not implicated in sexual dimorphism by itself.” (line160-169)

***Comment 7.*** *The section on Architecture of SIFa-positive neurons in the central nervous system does not add to the manuscript in my opinion, especially since it is difficult to make sense of the evidence presented in Figure S3.*
 **Answer:** Thank you for your feedback on our manuscript. We understand your concerns regarding the section on “Architecture of SIFa-positive neurons in the central nervous system” and we have carefully considered your comments.

We would like to emphasize that, although the architecture of SIFa-positive neurons has been previously described by Martelli et al [5] , Dick R Nässel [6] , our work provides novel and complementary insights. Specifically, we have utilized 3D reconstruction to precisely determine the 3D location of SIFa-positive neurons, revealing the spatial distribution of these neurons from the posterior to the anterior region. Furthermore, our analysis compares the neuronal processes of SIFa neurons across different sexes and developmental stages (adults vs. larvae), demonstrating that SIFa neuronal processes cover a significantly larger proportion of the brain in adults (approximately 40%) compared to larvae (only 10%).

By co-expressing *UAS-Denmark* and *UAS-syt.eGFP*, we have also identified the distinct distribution of dendrites and presynaptic terminals of SIFa neurons, showing that dendrites are confined to the central brain area while presynaptic terminals extend throughout nearly the entire brain region. These findings enhance our understanding of the functional roles of SIFa neurons within the neuronal circuitry and provide a more comprehensive view of their architecture.

***Comment 8.*** *"The Fly SCope single-cell RNA sequencing data indicates that SIFa neurons exhibit the highest level of co-expression for VGlut and ple (Fig 2N-2O)." Based on FlySCope it is not possible to determine the most highly co-expressed gene along with SIFa. You can only compare a maximum of 3 genes at a time.*

**Answer:** Thank you for your valuable feedback on our manuscript. We appreciate your comments regarding the FlyScope data and the limitations in determining the most highly co-expressed genes with SIFa.

We acknowledge that our initial statement may have been misleading and have revised it to better reflect the capabilities of the FlyScope tool. We agree with reviewer and changed sentence as below in main text.

“Fly Scope single-cell RNA sequencing data suggest potential high-level co-expression of *VGlut* and *ple* in SIFa neurons (Fig 2N-2O).” (line256-257)

This change in wording ensures that our claims are aligned with the technical constraints of the FlyScope platform, which allows comparison of a maximum of three genes at a time. We appreciate your guidance in helping us improve the accuracy of our presentation.

**Reference**

1. Inagaki HK, Ben-Tabou de-Leon S, Wong AM, Jagadish S, Ishimoto H, Barnea G, et al. Visualizing Neuromodulation In Vivo: TANGO-Mapping of Dopamine Signaling Reveals Appetite Control of Sugar Sensing. Cell. 2012;148: 583–595. doi:10.1016/j.cell.2011.12.022

2. Xie T, Ho MCW, Liu Q, Horiuchi W, Lin C-C, Task D, et al. A Genetic Toolkit for Dissecting Dopamine Circuit Function in Drosophila. Cell Rep. 2018;23: 652–665. doi:10.1016/j.celrep.2018.03.068

3. Shao J, Liewald JF, Costa WS, Ruse C, Gruber J, Djamshedzad MS, et al. Loss of neuropeptidergic regulation of cholinergic transmission induces homeostatic compensation in muscle cells to preserve synaptic strength. PLOS Biol. 2025;23: e3003171. doi:10.1371/journal.pbio.3003171

4. Watteyne J, Chudinova A, Ripoll-Sánchez L, Schafer WR, Beets I. Neuropeptide signaling network of Caenorhabditis elegans: from structure to behavior. GENETICS. 2024;228: iyae141. doi:10.1093/genetics/iyae141

5. Martelli C, Pech U, Kobbenbring S, Pauls D, Bahl B, Sommer MV, et al. SIFamide Translates Hunger Signals into Appetitive and Feeding Behavior in Drosophila. Cell Rep. 2017;20: 464–478. doi:10.1016/j.celrep.2017.06.043

6. Nässel DR, Zandawala M. Endocrine cybernetics: neuropeptides as molecular switches in behavioural decisions. Open Biol. 2022;12: 220174. doi:10.1098/rsob.220174
